# Faster flux of neurotransmitter glutamate during seizure — Evidence from ^13^C-enrichment of extracellular glutamate in kainate rat model

**DOI:** 10.1371/journal.pone.0174845

**Published:** 2017-04-12

**Authors:** Keiko Kanamori

**Affiliations:** Department of Epilepsy, Huntington Medical Research Institutes, Pasadena, California, United States of America; University of Modena and Reggio Emilia, ITALY

## Abstract

The objective is to examine how the flux of neurotransmitter glutamate from neurons to the extracellular fluid, as measured by the rate of ^13^C enrichment of extracellular glutamate (GLU_ECF_), changes in response to seizures in the kainate-induced rat model of temporal-lobe epilepsy. Following unilateral intrahippocampal injection of kainate, GLU_ECF_ was collected by microdialysis from the CA1/CA3 region of awake rats, in combination with EEG recording of chronic-phase recurrent seizures and intravenous infusion of [2,5-^13^C]glucose. The ^13^C enrichment of GLU_ECF_ C5 at ~ 10 picomol level was measured by gas-chromatography mass-spectrometry. The rate of ^13^C enrichment, expressed as the increase of the fractional enrichment/min, was 0.0029 ± 0.0001/min in frequently seizing rats (*n* = 4); this was significantly higher (*p* < 0.01) than in the control (0.00167 ± 0.0001/min; *n* = 6) or in rats with infrequent seizures (0.00172 ± 0.0001/min; *n* = 6). This result strongly suggests that the *flux* of the excitatory neurotransmitter from neurons to the extracellular fluid is significantly increased by frequent seizures. The extracellular [^12^C + ^13^C]glutamate *concentration* increased progressively in frequently seizing rats. Taken together, these results strongly suggest that the observed seizure-induced high flux of glutamate overstimulated glutamate receptors, which triggered a chain reaction of excitation in the CA3 recurrent glutamatergic networks. The rate of ^13^C enrichment of extracellular glutamine (GLN_ECF_) at C5 was 0.00299 ± 0.00027/min in frequently seizing rats, which was higher (*p* < 0.05) than in controls (0.00227 ± 0.00008/min). For the first time in vivo, this study examined the effects of epileptic seizures on *fluxes* of the neurotransmitter glutamate and its precursor glutamine in the *extracellular fluid* of the hippocampus. The advantages, limitations and the potential for improvement of this approach for pre-clinical and clinical studies of temporal-lobe epilepsy are discussed.

## Introduction

Glutamate excitotoxicity is thought to play an important role in the initiation and maintenance of epileptic seizures [1, 2]. This concept is based on evidence of significant elevation of glutamate in the extracellular fluid (GLU_ECF_) in the hippocampus of temporal-lobe epilepsy patients before or during complex partial seizure [1, 3], as well as higher GLU_ECF_ concentration in the inter-ictal period in the epileptogenic vs. non-epileptogenic hippocampus and in the atrophic vs. non-atrophic hippocampus [4–6]. However, the cause of GLU_ECF_ elevation—whether it is due to the accelerated release of neuronal GLU to synaptic fluid, or to the impaired uptake of GLU_ECF_ into glia, or to both—remains an open question [7, 8].

Numerous animal models of temporal lobe epilepsy have contributed to the advancement of knowledge in this area. Among these models, the chronic kainate-induced rodent model [9–13] most closely resembles human temporal lobe epilepsy in the EEG, morphological and biochemical abnormalities. Our previous study showed that the *concentration* of GLU_ECF_ is elevated in response to chronic-phase electrographic seizures [14], as well as in response to status epilepticus [3]. The concentration of GLU_ECF_ depends on the rate of neuronal GLU release to the extracellular fluid (ECF) relative to the rate of GLU_ECF_ uptake: when the rate of release is faster than the rate of uptake, which is known to be mainly into glia [15], the concentration is elevated. To examine the effect of seizure on the *rate of release* of neuronal GLU to ECF in vivo, it is necessary to perform isotopic labelling of neuronal GLU. The aim of the present study is to ^13^C-label brain GLU by intravenous infusion of [2,5-^13^C]glucose, in order to investigate whether *the rate of release of neuronal GLU to ECF* differs between seizing and non-seizing rats.

Previous ^13^C-labelling studies in temporal lobe epilepsy patients [16, 17] and in rodent models [18, 19] have focused on the effect of seizures on the ^13^C enrichment of GLU and other neurochemicals in hippocampal tissue, which is predominantly *intracellular*, using ^13^C NMR or gas-chromatography mass-spectrometry (GCMS) to quantify ^13^C-metabolites in brain extracts. To the best of our knowledge, there has been no human or animal study to examine the effect of seizures on the flux of neurotransmitter GLU from neuron to ECF, as measured by the rate of ^13^C enrichment of *extracellular* GLU (GLU_ECF_) during intravenous infusion of a ^13^C-labelled precursor. This is partly due to technical difficulty in measuring the ^13^C enrichments of picomol quantities of GLU_ECF_, because basal GLU_ECF_ concentration is only ~1/2,000^th^ of that of intracellular brain GLU [20]. However, the feasibility of analyzing the ^13^C-enrichment of GLU_ECF_ C5 (the side-chain carboxylate carbon) by GCMS was demonstrated in our previous work in the cortico-striatal region of normal rats [21]. For the first time in vivo, the present study shows how the flux of the excitatory neurotransmitter GLU from neuron to ECF is increased during frequent seizures in awake, freely behaving kainate-treated rats. This result, when combined with the time-course of change in the concentration of total [^12^C+^13^C]GLU_ECF_ measured by high-pressure liquid chromatography, suggests that the primary cause of GLU_ECF_ elevation in this group of rats is increased release of GLU to ECF that is associated with frequent electrographic seizures. The advantages, limitations and the potential for improvement of this ^13^C labelling approach for pre-clinical and clinical studies are discussed.

The metabolic and transport pathways of GLU in the brain compartments of neuron and glia are closely linked to those of glutamine (GLN) by the glutamine/glutamate cycle [22]. As shown schematically in S1 Fig, neurotransmitter GLU released from the synaptic vesicles to ECF is taken up mainly into glia by the excitatory amino acid transporter subtype 2 (EAAT2) [15] and metabolized to GLN by the glia-specific glutamine synthetase [23]. GLN is then transported to ECF by the sodium-coupled neutral amino acid transporter subtype 3 (SNAT3) [24]. GLN_ECF_ is taken up into neurons by the sodium-coupled neutral amino acid transporter subtypes 1 and 2 (SNAT 1 and 2) (reviewed by [25]), where it is hydrolyzed by glutaminase to replenish the metabolic and neurotransmitter pools of GLU. In view of this close precursor-product relationship of GLU and GLN and the emerging concept that epileptic seizures enhance neuronal uptake of GLN_ECF_ [14, 26], it was of interest to examine the time-course of the ^13^C enrichment of GLN_ECF_ C5 in addition of that of GLU_ECF_ C5.

## Materials and methods

### Chemicals

[2,5-^13^C]glucose (98% ^13^C) was purchased from Omicron Biochemicals Inc.(South Bend, IN, U.S.A). *N*-Methyl-*N*-(*t*-butyldimethylsilyl)-trifluoroacetamide (MTBSTFA) was purchased from Regis Technologies (Morton Grove, IL, U.S.A.).

### Kainic acid injection

All studies were approved by the HMRI Institutional Animal Care and Use Committee in accordance with the US Public Health Service’s Guide for the Care and Use of Laboratory Animals.

Adult male Wistar rats (240–260 g) were anesthetized with pentobarbital (40 mg/kg wt) and placed on a stereotaxic instrument. Kainic acid (KA) was injected unilaterally into the CA3 region of the right hippocampus at AP = -5.6 mm, L = + 4.5 mm and V = 5.5 mm [27]. Sodium kainate (Sigma-Aldrich, St. Louis, MO, USA) dissolved in 0.1 M phosphate buffer was injected with a 0.5 μL syringe at a dose of 0.4 μg/0.2 μL for a 325 g rat and adjusted according to body weight [14]; in other words, 0.295–0.32 μg was injected in a 240-260g rat. The rat, which awoke from anesthesia within 1 h, was continuously monitored for behavioral seizures for 6 h after injection (acute phase).

### Implantation of the EEG electrode and microdialysis guide cannula

At 43–49 days after KA injection, EEG electrodes (Plastics One, Roanoke, VA. USA) and microdialysis guide cannula with a stylet (Bioanalytical systems, West Lafayette, IN, USA) were implanted as described previously [14]. Briefly, the rat, which was anesthetized with ketamine/xylazine (100/5.2 mg/kg wt), was placed on the stereotaxic instrument and the skull was exposed. The grounding electrode (a stainless-steel wire 0.125 mm in diameter and 20 mm in length and terminating in a socket) was fixed to the interparietal bone with an anchor screw. The recording electrode (a pair of stainless steel wires of the same dimension with tips 0.5 mm apart) was attached (with Loctite 401) to the microdialysis guide cannula such that the electrode tips were 1.6 mm below the end of the guide cannula. This EEG electrode/microdialysis guide cannula complex was implanted bilaterally at coordinates of AP = -5.6 mm, L = ± 4.5 mm and V = 5.2 mm for the electrode and 3.6 mm for the end of the guide cannula. This places the electrode tip in CA3 region and the end of the microdialysis guide cannula at V = 3.6 mm from the skull surface, just above the CA1 region [27] as illustrated in S2 Fig (reproduced with permission from [28]). The recording electrodes and adjacent anchor screws were then fixed to the skull with acrylic cement. The sockets (one from the grounding electrode and two pairs from the recording electrodes) were inserted into the bottom contacts of a 6-pin plastic pedestal, which was then cemented to the skull and capped.

### EEG recording

Several days after the surgery, a preliminary EEG recording was taken. The lightly anesthetized rat was placed in RATURN (BioAnalytical Systems, West Lafayette, IN, U.S.A.) with its collar attached to a balance arm. The EEG electrode contacts on the skull were connected to a 90-cm cable, which was mesh-covered on the proximal end and equipped with solder lugs on the distal end for connection to the amplifier. The EEG cable was passed through an opening in the sensor of RATURN, which allows for EEG recording in freely behaving rats [26]. Recordings were taken wide-band 0.1 Hz to 1 KHz and sampled at 10 KHz/channel with a gain of 10K. Data were acquired and processed with DATAPAC 2K2 software (Run Technology, Mission Viejo, CA, USA). Preliminary EEG recordings were taken for several hours with the observation of behavioral seizures. When a rat developed EEG seizures characteristic of chronic-phase spontaneous seizures [12–14, 26], it was considered ready for the EEG/microdialysis/i.v. infusion experiment.

Two days before the experiment, an indwelling silastic catheter was placed in the right external jugular vein for i.v. infusion of glucose. The distal end of the catheter (90 cm long), exiting at the nape, was placed in a backpack worn by the rat [26].

### EEG/Microdialysis/i.v. infusion experiment

On the day of the experiment, the rat was lightly anesthetized with pentobarbital and placed in RATURN. The EEG cable, the inlet and outlet dialysis tubings and the i.v. infusion cannula were passed through an opening in its sensor, to permit the experiment in a freely behaving rat. Pentobarbital was used for consistency with our previous studies [14, 26]. The necessity of connecting the inlet and outlet tubings to the brain microdialysis probe precludes the use of isoflurane anesthesia for this experiment.

For microdialysis, the guide stylet was replaced with a microdialysis probe 320 μm in OD and 2 mm in length (Bioanalytical systems, West Lafayette IN, USA). This places the probe at V = 3.6 to 5.6 mm from the skull surface (S2 Fig). The probe was perfused at a rate of 2 μL/min with artificial cerebrospinal fluid (aCSF) containing the following equivalents of electrolytes (mM); 150 Na^+^, 3.0 K^+^, 1.4 Ca^2+^, 0.8 Mg^2+^, 1 PO_4_^3-^ and 155 Cl^-^ at pH 7.4. The probe collects extracellular fluid from hippocampal tissue ~700 μm in diameter and 2 mm in length. This location permits the collection of ECF, which optimally reflects GLU released from pyramidal neurons of the CA1 and CA3 regions affected by KA injection (V = 5.5 mm) without extensive dilution by ECF from unaffected tissue. A 3-h stabilization period was allowed before the start of dialysate collection for analysis. Dialysates were collected bilaterally in 15-min fractions for 1 h without i.v. infusion, then every 5 min with concomitant EEG recording and i.v. infusion of glucose for 3 h. Thus, the interval between pentobarbital injection and the start of ^13^C glucose infusion for enrichment analysis was approximately 4 h (1 h until waking, additional 2 h for the stabilization of extracellular neurochemicals and 1 h for basal dialysate collection). [2,5-^13^C]glucose was given per 250 g body weight as a bolus injection of 225 micromol followed by 150 micromol administered in exponentially decreasing quantities over the next 8 min. Subsequently, a constant infusion rate of 1.0 mmol/h was used for 3 h [29]. This protocol, initially developed by Fitzpatrick et al. [30], achieves a steady-state blood glucose concentration in 9 min and brain glucose concentration in 30 min [29]. For controls, the same procedure was applied to normal rats given an injection of saline instead of kainic acid. Dialysates were frozen immediately and stored at -20°C until analysis. In KA rats, only dialysates collected from the KA-injected ipsilateral hippocampus were analyzed for total GLU_ECF_, GLN_ECF_, and for their ^13^C enrichments. In controls where the two hippocampi are equivalent, dialysate from each hippocampus was analyzed separately, with *n* referring to the number of hippocampi (2/rat).

After the experiment, the locations of the microdialysis probe and the electrodes were confirmed in each rat as described previously [14]. Briefly, the probe was removed and the guide stylet was reinserted. The cement fixing the guide cannula and the EEG electrodes to the skull could be lifted from the skull of the anesthetized rat by inserting a flat spatula between the cement and the nasal bone. Accordingly, the cement with the electrodes and the guide cannula still attached could be examined for vertical coordinates in every rat. The lateral coordinates, too, could be confirmed from the distance between the right and left electrodes and the location of the burr holes on the exposed skull. The confirmed coordinates of the microdialysis probe and the EEG electrode are shown in Results. The brain was then removed from the anesthetized rat and frozen in liquid nitrogen. This procedure, instead of fixing the brain for histology, was adapted at the start of the project because, in our previous studies on the same model, it was informative to measure the *intracellular* glutamine concentration in the relevant hippocampal region of the end-point brain for interpretation of the effect of seizure on GLN_ECF_ [14, 26]. However, the frozen brain was not used for this purpose in the present study, because the time-course of ^13^C enrichment of extracellular GLN, unlike that of extracellular GLU, turned out to show little dependence on seizure activity as described in Results.

### Identification of seizure

EEG seizure was identified according to the definition of [13] as a period of consistent and repetitive changes in amplitude and frequency of electrical activity that was clearly different from inter-ictal activity and that persisted for >10 s. In correlating EEG activity with changes in GLU_ECF_ and GLN_ECF_, the fact that it takes 180 s for dialysate to flow from the rat brain to the collection vial under these experimental conditions was taken into account. The microdialysis time was reported at the center of each collection time.

### HPLC assay of extracellular GLU and GLN

Amino acids in the brain dialysate were assayed after precolumn derivatization with ortho-phthaldehyde (OPA) and 2-mercaptoethanol and separation on a reverse-phase column by fluorometric detection as described previously [26].

### Purification and derivatization of GLU_ECF_ and GLN_ECF_ for GCMS

After a 2- or 3-μL aliquot of the dialysate was used for HPLC assay of GLU_ECF_ and GLN_ECF_, the remaining dialysates were pooled from two or three consecutive 5-min fractions, and partially purified by ion-exchange chromatography as described previously [21]. Briefly, the dialysate (50 ~75 μL) was loaded onto an AG1 column (Na^+^ form; 0.5 cm diameter X 2.4 cm height). After eluting neutral and cationic metabolites with water (1.8 ml), acidic metabolites, including GLU_ECF_, were eluted with 1 N HCl, frozen immediately in liquid nitrogen and freeze-dried to remove water and HCl. Meticulous care was taken to clean the column and the resin and all glassware to avoid contamination by trace [^12^C]GLU, as described in detail previously [21]. HPLC-grade water (Sigma Aldrich, St. Louis, MO, U.S.A.) was used for all experiments and cleaning. The lyophilized dialysate containing GLU_ECF_ was reconstituted in water (30 μL), transferred to the glass insert to be used for GCMS and dried in a vacufuge (Model 5301, Eppendorf, Hauppange, N.Y., U.S.A.) at 60°C for 4 h. The dried GLU_ECF_ was dissolved in anhydrous acetonitrile (6 μL) by sonication, by placing the glass insert fitted into a capped Eppendorf tube, in a sonic bath for 15 min.

The eluted GLN_ECF_ in the neutral fraction of the anion-exchange column was further purified by cation-exchange chromatography. After adjusting the pH to 2.0 at which GLN_ECF_ has a net positive charge, the fraction was loaded onto an AG50 column (H^+^ form; 0.5 cm diameter x 1 cm height) and washed with water (1.5 ml) to remove neutral metabolites. GLN_ECF_ was then eluted with 1 ml of 1N NH_4_OH, frozen immediately in liquid nitrogen and freeze-dried for the removal of water and NH_3_.

GLU_ECF_ in acetonitrile was derivatized by the addition of 1.5 μL of *N*-Methyl-*N*-(*t*-butyldimethylsilyl)-trifluoroacetamide (MTBSTFA) and incubation overnight at 25°C. For GLN_ECF_, a different approach was taken because reports from other laboratories [31, 32] and our experience showed that *t*BDMS-glutamine was unstable. Accordingly, glutamine was converted to pyroglutamic acid, which is a cyclic compound that contains all five carbons of glutamine and forms a very stable *t*BDMS-derivative [32]. To convert glutamine to pyroglutamate with loss of NH_3_, the dried dialysate containing GLN_ECF_ was reconstituted with 250 μL of water and heated at 100°C for 3 h in a capped vial in a block heater. The pH was then adjusted to 2.0 to convert pyroglutamate to pyroglutamic acid for derivatization. It is to be noted that endogenous pyroglutamate in ECF, which exhibits a concentration of 17 μM in normal human CSF [33], has a net negative charge at physiological pH. Accordingly, it binds to the AG1 resin described above and is completely separated from eluted GLN_ECF_ in the neutral fraction. Thus, the GLN_ECF_-derived pyroglutamic acid used for ^13^C enrichment analysis is quite free of endogenous pyroglutamic acid. GLN_ECF_-derived pyroglutamic acid was dried in a vacufuge at 60°C for 3 h, dissolved in acetonitrile (10 μL), and derivatized with MTBSTFA (2 μL) by incubation overnight at 25°C. Derivatization, which is optimized under anhydrous conditions, was carried out in a glove bag filled with dry nitrogen gas to maintain the relative humidity below 20%.

### ^13^C enrichment analyses by GCMS

Separation of *t*BDMS-metabolites and ^13^C enrichment analyses were performed on Hewlett-Packard 6890GC-5973MSD or 5890GC-5972MSD, located in the Global Environmental Analyses Center at California Institute of Technology. Separation was achieved on a 30 m x 0.25 mm x 0.25 μm HP-5MS capillary column with the following oven-temperature program: 65°C for 1.3 min, then 25°C/min to 240°C (0) then 10°C/min to 300°C (3 min). The injector temperature was 275°C. 1 μl of sample was injected in the automated splitless mode. For MS, the operating conditions were 70 eV electron-impact ionization and ^13^C enrichment analyses by selected ion monitoring. Two GCMS experiments were performed on a Waters GCT Premier Instrument, equipped with a ZB5 column (30 m x 0.25 mm x 0.25 μm) and a time-of-flight mass spectrometer.

For *t*BDMS-GLU, selected ion monitoring was performed at three ion pairs, m/z 272/273 and 330/331 (both of which contain C2-C5 of GLU) and m/z 432/433 (which contains C1-C5). The structures of the fragment ions are shown in Results.

For glutamine-derived *t*BDMS-pyroglutamic acid, selected ion monitoring was performed at ion pairs m/z 198/199 and m/z 272/273 (both of which contain C2-C5 of glutamine) and at m/z 330/331 which contains C1-C5 of glutamine. The injection liner and the septum were changed frequently and the injection needle was rinsed 12 times with acetonitrile after each injection. Between each biological sample, a blank consisting of the solvent acetonitrile and MTBSTFA was run to ensure no carry-over of ^13^C enriched derivative.

Biological ^13^C enrichment resulting from i.v. infusion of [2,5-^13^C]glucose was calculated after subtracting the contribution from naturally occurring heavy isotopes, as shown below for the m/z 331/330 ion pair of *t*DMBD-GLU. The fragment ion m/z 330 has a composition of C_16_H_21_O_2_Si_2_N_1_. Hence, the contribution of the naturally occurring heavy isotope of each element to the signal at m/z 331 is 27.8%. This is experimentally verified by the observed peak area ratio of the m/z 331 to m/z 330 ion in the mass spectrum of non-^13^C enriched *t*BDMS-GLU. Correction for the contribution of naturally occurring heavy isotopes was performed as follows. Let A be the observed peak area of the signal at m/z 331 and B the observed peak area of the signal at m/z 330. Then, the observed fractional biological ^13^C enrichment of this ion pair is given by (A/B − C)/[1 + (A/B − C)] where C = 0.278 is the correction factor for the contribution of the naturally occurring heavy isotopes. The fractional enrichment determined by this method provides accurate biological ^13^C enrichment at C2 to C5 of GLU contained in the m/z 331/330 ion fragment [21]. The corresponding correction factors were 0.362 (m/z 433/432), 0.278 (m/z 331/330), and 0.234 (m/z 273/272) for ion pairs of *t*BDMS-GLU, and for the ion pairs of *t*BDMS-pyroGLU, they were 0.245 (m/z 301/300), 0.234 (m/z 273/272) and 0.161 (m/z199/198). For ^13^C enrichment at C2-C5 of *t*DMBD-GLU, the mean enrichment determined from ion pairs m/z 331/330 and m/z 273/272 (which were within ±1%) was reported. Similarly, for ^13^C enrichment at C2-C5 of *t*DMBD-pyroGLU, the mean enrichment obtained from the ion pairs m/z 273/272 and m/z199/198 were reported.

### Calculation of ^13^C enrichment at C5

In this study, the expression ^13^C *enrichment* (instead of ^13^C *labelling)* is used to denote enrichment above the natural abundance (1.1%) of ^13^C, for consistency with our previous publication [21]. In publications from other laboratories where “labelling” is used in the text, quantitative data are always reported as percentage enrichment (for example [16–19]). Our previous results showed that i.v. infusion of [2,5-^13^C]glucose significantly enriches brain GLU only at C5 and to a lesser extent at C1, while enrichment at C2, C3, C4 was very low [29]. To quantify the low enrichment at C2, C3 and C4 relative to the enrichment at C5, ^13^C NMR spectra were taken of the perchloric acid extracts of the brain after 2 and 3.6 h of [2,5-^13^C]glucose infusion at 50 MHz for ^13^C on a Bruker-GE CSI-II spectrometer using a solenoidal ^13^C probe combined with a saddle-type ^1^H coil for shimming and decoupling. The results are shown in S1 Table (“Distribution of ^13^C in C2-C5 of GLU and GLN after intravenous infusion of [2,5-^13^C]glucose as measured in brain extract by ^13^C NMR”). The enrichment data in the third column were used to calculate the ratio of ^13^C enrichment at C2+C3+C4 to the enrichment at C2+C3+C4+C5 (the last column). For GLU, this ratio was only 0.098 at *t* = 2 h and 0.198 at *t* = 3.6 h. As shown in S3A Fig, this ratio increased linearly with time with a slope of 0.00089. This slope was used to calculate the enrichment at C2+C3+C4 at each time point of [2,5-^13^C]glucose infusion and was subtracted from the ^13^C enrichment at C2+C3+C4+C5 observed in the *t*BDMS-GLU of biological samples by MS to obtain the ^13^C enrichment at C5 reported in this study.

For GLN, as shown in S1 Table, this ratio is higher than for GLU at *t* = 2 h, reflecting the fact that ^13^C labeling at C3 and C2 occurs by the glia-specific pyruvate carboxylase pathway [34, 35]. The increase was biphasic with a small further increase at 3.6 h (S3B Fig). Accordingly, the enrichment at C2+C3+C4 was calculated from the slope 0.0016 for *t* = 0–2 h, and the slope 0.00042 was used to calculate additional enrichment between t = 2–3 h; these enrichments were subtracted from the observed ^13^C enrichment at C2+C3+C4+C5 of *t*BDMS-pyroGLU to obtain the ^13^C enrichment at GLN_ECF_ C5 reported in this study. The validity of this approach in relation to reports from other laboratories is discussed in Discussion.

### Statistical analyses

Single-factor ANOVA was used to examine whether the observed mean value (e.g. mean GLU_ECF_) differed significantly among the three groups, viz frequently seizing KA rats (group III), infrequently seizing KA rats (group II) and controls (group I). F values used to establish significant differences are reported, in addition to *p*-values with a significant difference at *p* < 0.05 shown by * and that at *p* < 0.01 by ** in figures. Data analyses, including post-hoc tests to determine which group, among the three, differs significantly from another, were performed by the statistical software of QI Macros (KnowWare International Inc., Denver, CO, USA), which indicates whether the significant difference was established by the Scheffe’s test or by the Tukey’s HSD test, as specified for each *p* value in the Result.

## Results

### EEG characteristics of KA rats

Upon unilateral KA injection in the hippocampal CA3 region, all rats developed acute-phase behavioral seizures corresponding to stages 1–5 of Racine’s classification [36] lasting up to 6 h. At the low dose of kainate, 0.295–0.32 μg injected in a 240-260g rat, there was no mortality during the acute or the subsequent latent and chronic phases. Preliminary EEG recordings in the chronic phase showed that 60% of the KA rats from a total of 15 exhibited recurrent electrographic seizures during several hours of recording. Those 10 rats were chosen for EEG/microdialysis/^13^C-glucose infusion experiments.

As shown in Table 1, those kainate-treated (KA) rats were examined 49–56 days after unilateral kainate injection. The numbers of spontaneous recurrent seizures that occurred during 3 hours of glucose infusion are shown. Those KA rats fell into two groups: those that showed infrequent intermittent seizures (mean ± SE of 5.0 ± 0.37) and those that showed frequent seizures (10 ± 0.7). In this study, controls were designated as group I, KA rats with infrequent seizures as group II and those with frequent seizures as group III. As shown in Table 1, the locations of the EEG electrodes and microdialysis probes, implanted stereotaxically and confirmed at the end of in vivo experiments, were very close.

**Table 1:** EEG characteristics, coordinates of EEG electrodes and microdialysis probes and basal GLU_ECF_ and GLN_ECF_ concentrations in control and kainate-injected rats.

Rat group	Days post-KA	Seizure (# in 3 h)	Coordinates (mm)					Pre-infusion dialysate concentration (μM)	
			EEG electrode			Microdialysis probe			
			AP	Lateral	Vertical	Vertical		GLU_ECF_	GLN_ECF_
						top	bottom		
I. Control (*n* = 6)	n.a.	none	-5.6	4.55 ± 0.03	5.45 ± 0.17	3.7 ± 0.21	5.7 ± 0.21	1.27 ± 0.43	29.9 ± 3.5
II. KA (infrequent seizures) (*n* = 6)	49.5 ± 1.0	5.0 ± 0.37	-5.6	4.57 ± 0.08	5.3 ± 0.19	3.6 ± 0.24	5.6 ± 0.24	0.94 ± 0.069	36.4 ± 3.0
III. KA (frequent seizures) (*n* = 4)	55.8 ± 3.7	10 ± 0.7	-5.6	4.58 ± 0.06	5.20 ± 0.24	3.6 ± 0.22	5.6 ± 0.22	2.13 ± 1.0	29.5 ± 2.7

**Fig 1:**
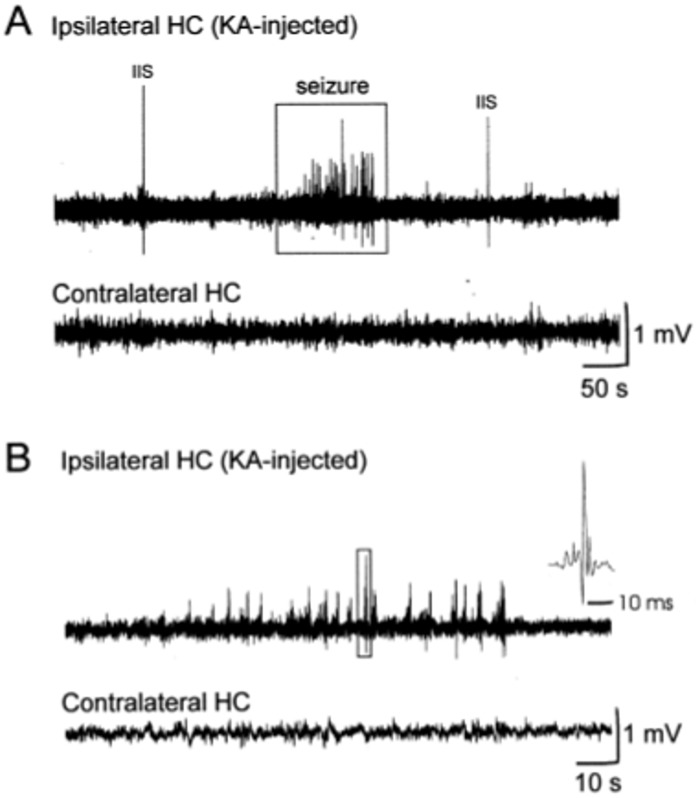
EEG recording of a chronic-phase seizure in a freely behaving KA rat. **(A)***Top*: The kainate-injected ipsilateral hippocampus shows a quiescent period with a single inter-ictal spike (IIS) followed by a seizure (enclosed in box). *Bottom*: The contralateral hippocampus was seizure-free. **(B)** *Top*: Expanded plot of the seizure with a time-scale of 10 s. The inset shows an expanded plot of the peak in the box with a time scale of 10 ms; the wave pattern is characteristic of a hypersynchronous population burst from glutamatergic neurons. *Bottom*: Corresponding recording from the seizure-free contralateral hippocampus.

Fig 1 shows EEG recordings from the hippocampi of an awake freely behaving KA rat (R1125 from the frequently seizing group) during the chronic phase. The top trace of Fig 1A shows a recording from the kainate-injected ipsilateral hippocampus. A quiescent period characterized by a single inter-ictal spike (IIS) is followed by a seizure (Box), which, by definition (see section **Identification of seizure**), is a period of consistent and repetitive changes in amplitude and frequency of electrical activity that is clearly different from inter-ictal activity. This seizure occurred in the ipsilateral hippocampus (top) but was absent from the contralateral hippocampus (bottom). This is characteristic of a hypersynchronous onset seizure [12, 13], in which behavioral components are absent or mild (stages 1–3 of Racine’s classification [36]). Fig 1B top shows an expanded plot of the seizure, with a time-scale of 10 s. The inset shows an expanded plot of the peak in the box that shows a wave pattern characteristic of a population burst from glutamatergic neurons [37]. For the seizures listed in Table 1, hypersynchronous onset seizures represented 74% and the remaining 26% were low-voltage fast onset seizures accompanied by occasional motor clonus (stage 4 of Racine’s classification [36]). The occurrence of these two types of spontaneous seizures, with the hypersynchronous onset as the major type, during the chronic phase of KA rats, is consistent with a previous report [13].

### ^13^C enrichment of GLU_ECF_ was significantly faster in frequently seizing rats

**Fig 2:**
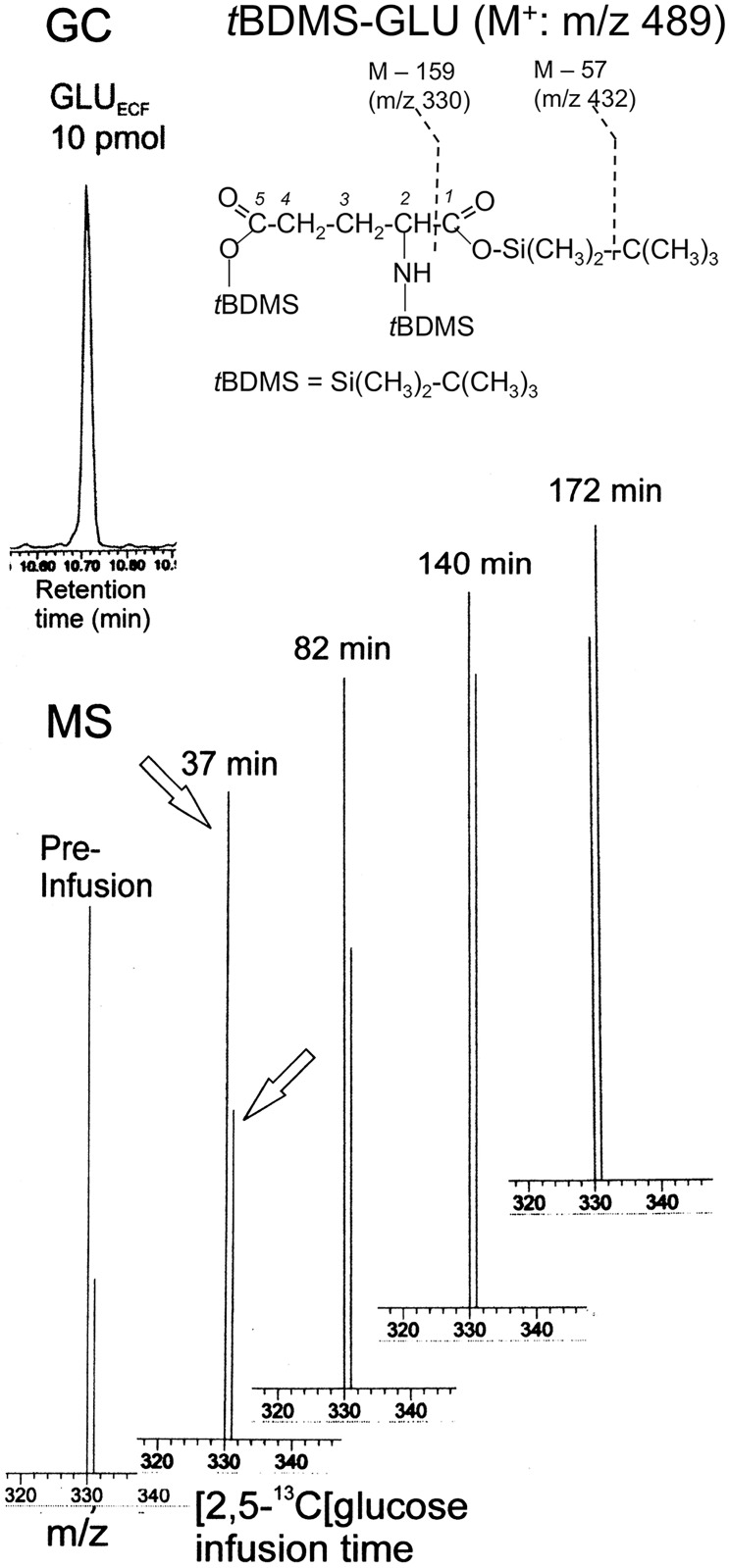
Progressive ^13^C enrichment of GLU_ECF_ during i.v. infusion of [2,5-^13^C]glucose, as observed by GCMS. The top left panel shows a GC chromatogram of 10 picomol of *t*BDMS-GLU. On the right are shown the structures of *t*BDMS-GLU and of the fragment ions m/z 432 and m/z 330. The loss of another *t*-butyl group from the latter gives rise to the m/z 272 ion. The bottom panel shows representative mass spectra of the ion pair m/z 330/331 of the *t*BDMS derivative of GLU_ECF_ (which contain C2-C5 of GLU_ECF_) collected from the ipsilateral hippocampus of an epileptic rat (R1125 from group III) at the indicated time points during the i.v. infusion of [2,5-^13^C]glucose. A progressive increase in ^13^C enrichment was observed from the increase in the peak area of m/z 331 ion relative to that of the m/z 330 ion, shown by the arrows.

Fig 2 shows a GC chromatogram of 10 pmol of *t*BDMS-GLU (top left) and representative mass spectra (bottom) of *t*BDMS-derivative of GLU_ECF_ (structure at right top; see below for explanation) collected from the ipsilateral hippocampus of an epileptic rat (R1125 from group III) at the indicated time points during i.v. infusion of [2,5-^13^C]glucose. Rat brain GLU, including the neurotransmitter GLU released to ECF, can be ^13^C-enriched predominantly at C5, and to a lesser extent at C1, by intravenous infusion of [2,5-^13^C]glucose ([29] and references cited therein). GLU released into ECF is a highly polar compound which must be derivatized to a volatile compound, such as *t*BDMS-GLU, for separation by gas chromatography. Electron-impact ionization of *t*BDMS-GLU in the mass spectrometer produces three major ion fragments, m/z 432 (which contains C1-C5 of glutamate), and m/z 330 and m/z 272 (both of which contain C2-C5 of glutamate), as shown in Fig 2. Biological ^13^C enrichment increases the abundance of the m/z 331 ion (which is one mass unit heavier than the m/z 330 ion). Hence, the increase in ^13^C enrichment can be determined from the increase in the peak area of the m/z 331 ion relative to that of the m/z 330 ion (Methods for precise calculation of the ^13^C enrichment of GLU_ECF_ C5 are described in sections ^**13**^**C enrichment analyses by GCMS** and **Calculation of** ^**13**^**C enrichment at C5**). As shown in the mass spectra, a progressive increase in the ^13^C enrichment, compared to the pre-infusion level, was observed from the increase in the peak area of the m/z 331 ion relative to that of the m/z 330 ion.

**Fig 3:**
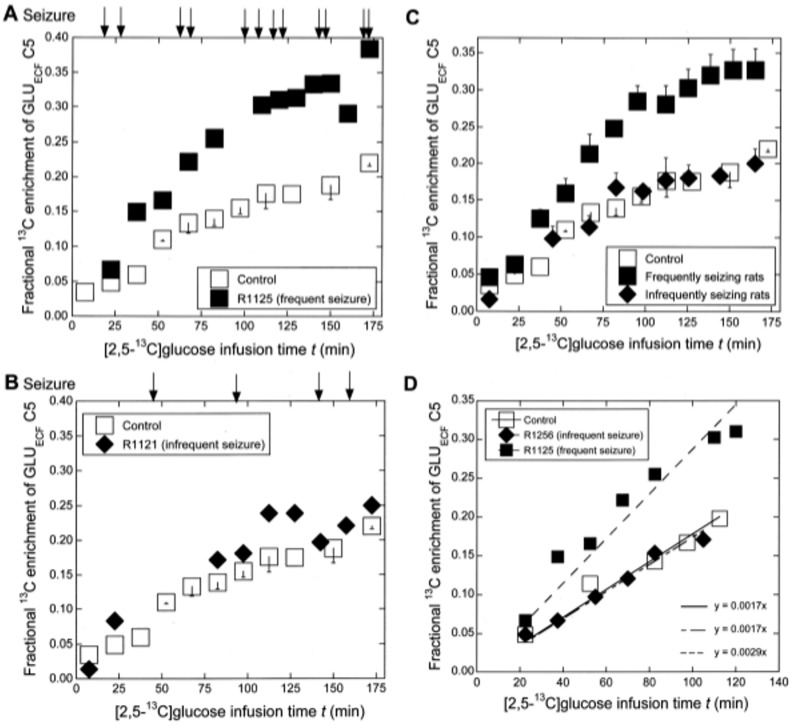
The time course of ^13^C enrichment of GLU_ECF_ C5. **A.** A representative time course in a frequently seizing KA rat (R1125: black square) compared to control (open square). In this rat, the seizures occurred at the times shown by arrows. The mean ± SE of the number of seizures in the frequently seizing KA rats is provided in Table 1, while the mean time course of the ^13^C enrichment of GLU_ECF_ C5 for this group is shown in Fig 3C. **B.** Time course (black diamond) in a representative KA rat with rare seizures (R1121; shown by arrows) compared to control (open square). The mean ± SE of the number of seizures in the infrequently seizing KA rats is provided in Table 1, while the mean time course of the ^13^C enrichment of GLU_ECF_ C5 in this group is shown in Fig 3C. **C.** Time course shown as the mean ± SE for controls (*n* = 6), infrequently seizing rats (*n* = 6) and frequently seizing rats (*n* = 4). The ^13^C enrichment was substantially faster in frequently seizing rats compared to both the control and the infrequently seizing rats. **D.** The least-squares fit for the calculation of the rate of ^13^C enrichment for *t* = 0–120 min for a representative rat from control, an infrequently seizing (R1256), and a frequently seizing (R1125) rat. The mean ± SE values of the rates of ^13^C enrichment of GLU_ECF_ C5 for each group are shown in Table 2.

Fig 3A shows the time course of ^13^C enrichment of GLU_ECF_ C5 in R1125, calculated from the observed enrichment of *t*BDMS-GLU in Fig 2 as described in Section **Calculation of** ^**13**^**C enrichment at C5**. The time of occurrence of seizures is shown by arrows at the top. ^13^C enrichment was faster in this frequently seizing rat compared to controls. Fig 3B shows the corresponding time course in one rat (R1121 from group II) that showed intermittent and fewer seizures at the times indicated by arrows. When seizures were rare, the time course of ^13^C enrichment of GLU_ECF_ C5 was similar to that in controls. Fig 3C compares the time course of ^13^C enrichment of GLU_ECF_ C5 as the mean ± SE for frequently seizing rats (*n* = 4) and infrequently seizing rats (*n* = 6) compared to controls (*n* = 6). The mean time course of ^13^C enrichment was substantially faster in frequently seizing rats compared to those in control or in infrequently seizing rats. The rate of ^13^C enrichment of GLU_ECF_ C5, expressed as the increase in fractional ^13^C enrichment/min, was calculated from the slope of the least-squares line through the plots for 0–120 min of [2,5-^13^C]glucose infusion for each rat. Fig 3D shows an example of the least squares line through the plots for a rat from each group; the slope is similar for the control and the infrequently seizing rat, but substantially higher for the frequently seizing rat. Then, from the rates of individual rats in each group, the mean rate for group I (control), group II (infrequently seizing KA rat) and group III (frequently seizing KA rats) was calculated. These rates are shown in Table 2 (the middle column). The rate in frequently seizing rats, 0.0029 ± 0.0001/min, was significantly higher than the rate in the control, 0.00167 ± 0.0001/min and also higher than the rate in infrequently seizing rats, 0.00172 ± 0.0001/min, with *p* < 0.01 (F = 27.0; Scheffe’s test).

**Table 2:** Rates of ^13^C enrichments of GLU_ECF_ C5 and GLN_ECF_ C5 during [2,5-^13^C]glucose infusion in control and KA rats undergoing infrequent or frequent seizures.

Rat group	The rate of ^13^C enrichment of GLU_ECF_ C5 (increase in fractional enrichment/min) during 0–120 min of [2,5-^13^C]glucose infusion	The rate of ^13^C enrichment of GLN_ECF_ C5 (increase in fractional enrichment/min) during 0–110 min of [2,5-^13^C]glucose infusion
I. Controls (*n* = 6)	0.00167 ± 0.0001	0.00227 ± 0.00008
II. Rats with infrequent seizures (*n* = 6)	0.00172 ± 0.0001	0.00265 ± 0.00020
III. Rats with frequent seizures (*n* = 4)	0.0029 ± 0.0001^a^	0.00299 ± 0.00027^b^

^*a*.^ Significantly higher than I and II.

^*b*.^ Significantly higher than I.

### Total [^12^C + ^13^C]GLU_ECF_ increases in frequently seizing rats

**Fig 4:**
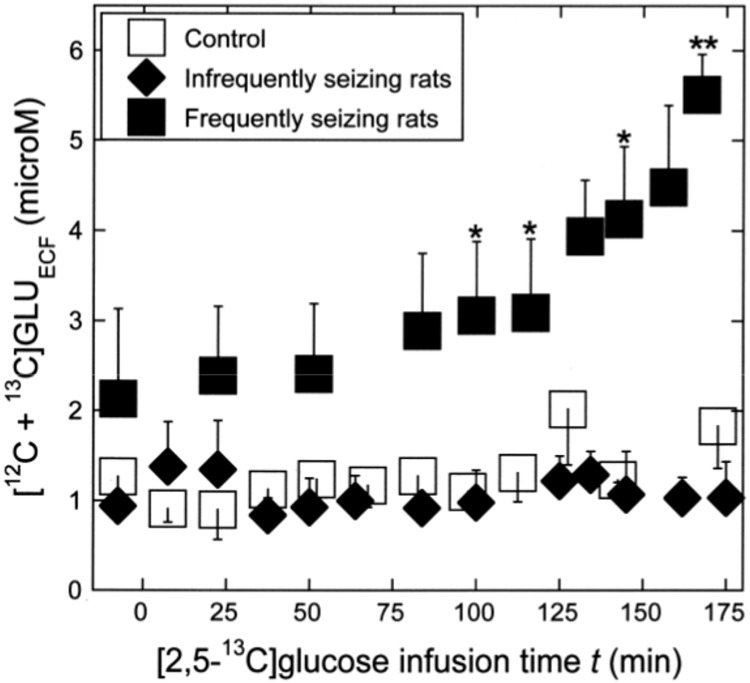
The time course of total [^12^C + ^13^C]GLU_ECF_. Total GLU_ECF_ showed little changes in the control (open square) or in infrequently seizing rats (black diamond), but in frequently seizing rats (black square) showed gradual elevation between *t* = 80–120 min and a significant elevation between *t* = 125–167 min (Fig 4). See text for the explanation of the significant differences at *t* = 97, 117, 144 and 167 min. Likely explanations for the observed multi-phase increases in the frequently seizing KA rats are described in Discussion.

As shown in Table 1, the pre-infusion concentrations of [^12^C + ^13^C]GLU_ECF_ were 1.27 ± 0.43 μM in controls, 0.94 ± 0.069 μM in infrequently seizing rats, and somewhat higher 2.13 ± 1.0 μM in frequently seizing rats, although there was no statistically significant difference among them. Fig 4 shows the time course of total [^12^C + ^13^C]GLU_ECF_ concentration in dialysate measured by HPLC. Total GLU_ECF_ showed little changes in the control (open square) or in infrequently seizing rats (black diamond), but, in frequently seizing rats (black square), it showed gradual elevation between *t* = 80–120 min and a significant further elevation between *t* = 125–167 min (Fig 4).

Although there was considerable scatter in the mean GLU_ECF_ concentration of the frequently seizing rats, the mean concentrations at *t* = 97 min, 117 min, and 144 min differed significantly from the concentrations in controls and from those in infrequently seizing rats at similar time points (*p* < 0.05), with F values of 6.0 (Tukey’s HSD test), 4.3 (Tukey’s HSD test) and 8.2 (Scheffe’s test) respectively. At the end of infusion (*t* = 167 min), the mean concentration of 5.51 ± 0.45 μM in frequently seizing rats differed significantly from that in control, 1.73 ± 0.29 μM, and from that in infrequently seizing rats,1.04 ± 0.4 μM, with *p* < 0.01 (F = 35.9; Scheffe’s test).

### ^13^C enrichment of GLN_ECF_

**Fig 5:**
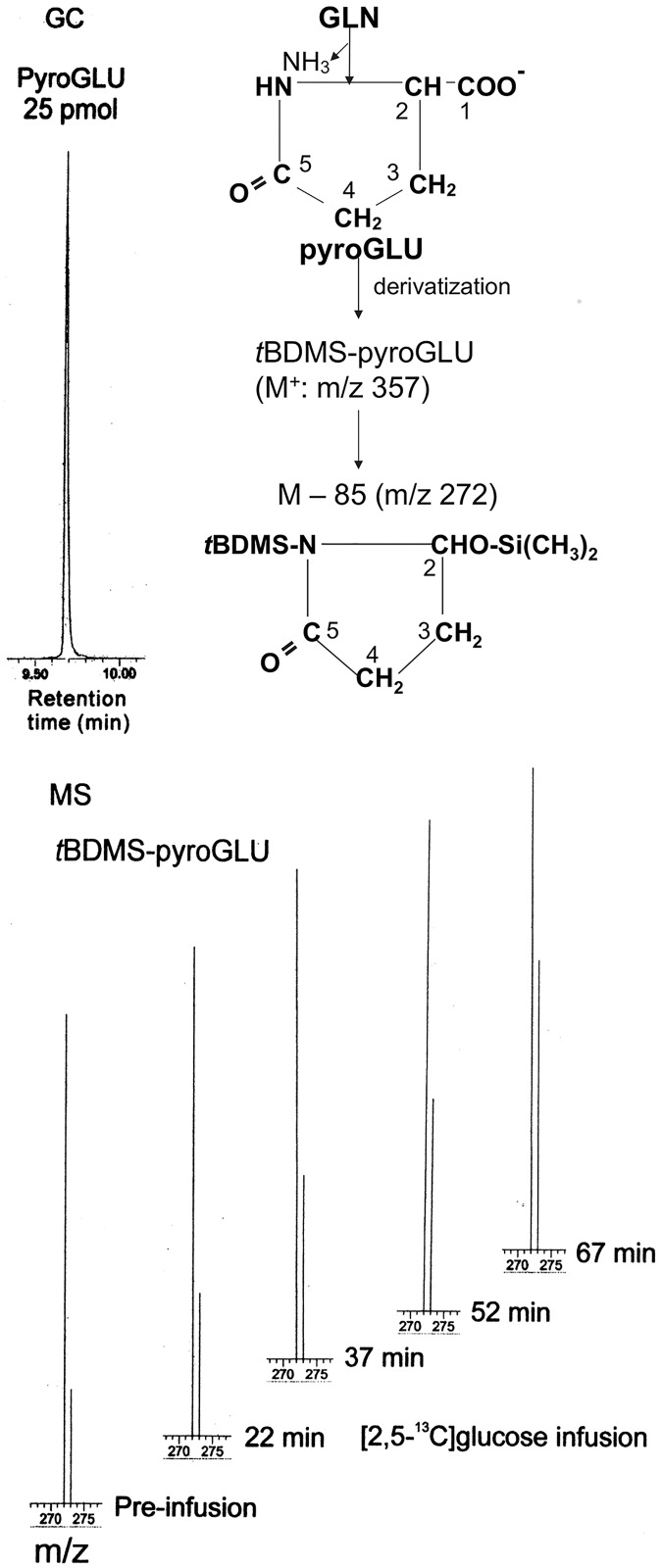
Progressive ^13^C enrichment of GLN_ECF_ during i.v. infusion of [2,5-^13^C]glucose, as observed by GCMS. *Right panel*: GLN_ECF_ was converted to pyroGLU and derivatized to *t*BDMS-pyroGLU, which, upon electron-impact ionization, produced fragment ions m/z 300, m/z 272 and m/z 198 as shown in S4 Fig. *Left panel*: GC chromatogram of 25 pmol of *t*BDMS-pyroGLU. *Bottom panel*: mass spectra of *t*BDMS-pyroGLU derived from GLN_ECF_ collected from the ipsilateral hippocampus during i.v. infusion of [2,5-^13^C]glucose. Progressive ^13^C enrichment was observed from the increase in the peak area of the m/z 273 ion relative to that of m/z 272 ion. The structure of the fragment ion with m/z 272 is shown at the bottom of the right panel.

As shown in Fig 5 (right panel), GLN_ECF_ was converted by loss of NH_3_ to pyroglutamic acid (pyroGLU), which contains all carbons of glutamine and forms a stable *t*BDMS derivative (Section **Purification and derivatization of GLU**_**ECF**_ **and GLN**_**ECF**_ **for GCMS**). The structures of *t*BDMS-pyroGLU (M+:m/z 357), and of its fragment ions m/z 300, m/z 272 and m/z 198 as described by [32] are shown in S4 Fig. An important fragment ion is the one with m/z 272 whose structure is shown in Fig 5 at the bottom of the right panel. The ion pairs m/z 272/273 and m/z 198/199, which contain C2 to C5 of glutamine, were used for ^13^C enrichment analyses. Fig 5 (top left) shows a GC chromatogram of 25 picomol of pyroGLU. At the bottom are shown representative mass spectra of *t*BDMS-pyroGLU derived from extracellular glutamine (GLN_ECF_), which was collected by microdialysis from the ipsilateral hippocampus of a frequently seizing rat (R1081) during intravenous infusion of [2,5-^13^C]glucose. Progressive increases in the ^13^C enrichment of C2 to C5 of GLN_ECF_ were observed from the increase in the peak area of the m/z 273 ion relative to that of the m/z 272 ion. The ^13^C enrichment at GLN_ECF_C5 was determined by subtracting the minor ^13^C-enrichment contributions of C2, C3 and C4 as described in Section **Calculation of** ^**13**^**C enrichment at C5**.

**Fig 6:**
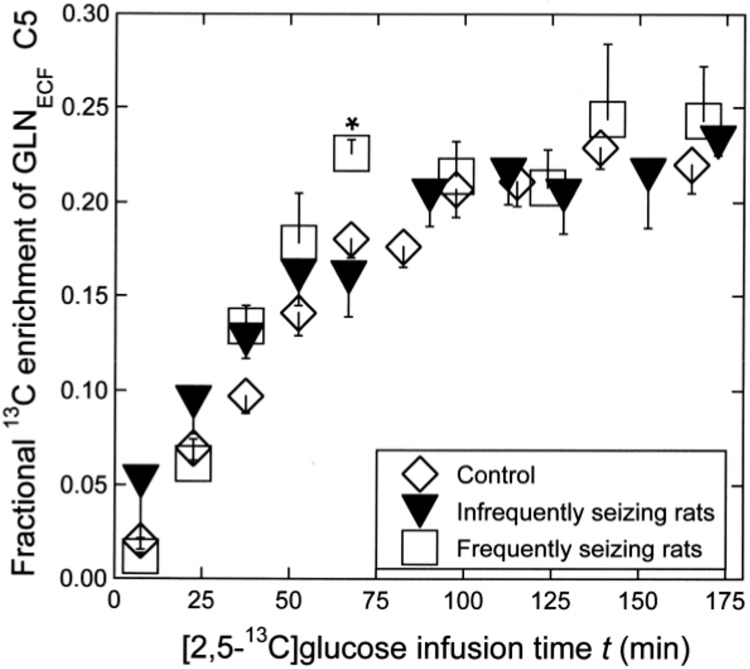
Time course of ^13^C enrichment of GLN_ECF_. Time course shown as the mean ± SE for infrequently seizing rats (black inverted triangle) and frequently seizing rats (open square) compared to the control (open diamond). The error bar, when invisible, is smaller than the symbol. See text for the explanation of the significant difference at *t* = 67 min. The mean ± SE values of the rates of ^13^C enrichment of GLN_ECF_ C5 over *t* = 0–110 min in the three groups are shown in Table 2, as explained in the text.

Fig 6 shows the time courses of ^13^C enrichment of GLN_ECF_ C5 (the mean ± SE) in infrequently and frequently seizing rats compared to the control. The time courses were very similar among the three groups, but in the frequently seizing rats, the mean enrichment of 0.23 ± 0.01 at *t* = 67 min was higher than in controls (0.17 ± 0.010) or in infrequently seizing rats (0.16 ± 0.021) with *p* < 0.05 (F = 5.2; Tukey’s HSD test). In the control and infrequently seizing rats, the ^13^C enrichment increased linearly up to 110 min then levelled off. Accordingly, the rate of ^13^C enrichment of GLN_ECF_ C5 was calculated from the slope of the least-square line through plots over this time interval in each rat, and the mean ± SE was taken for each group. The results are shown in Table 2 (last column). The rate of ^13^C enrichment of GLN_ECF_ C5 in frequently seizing rats, 0.00299 ± 0.00027/min, was significantly higher than the rate in control (0.00227 ± 0.00008/min) with *p* < 0.05 (F = 4.3; Scheffe’s test). The rate in infrequently seizing rats (0.00265 ± 0.0002/min) was not significantly different from the rate in the other two groups.

### Total [^12^C+^13^C]GLN_ECF_ decreases in response to seizure

**Fig 7:**
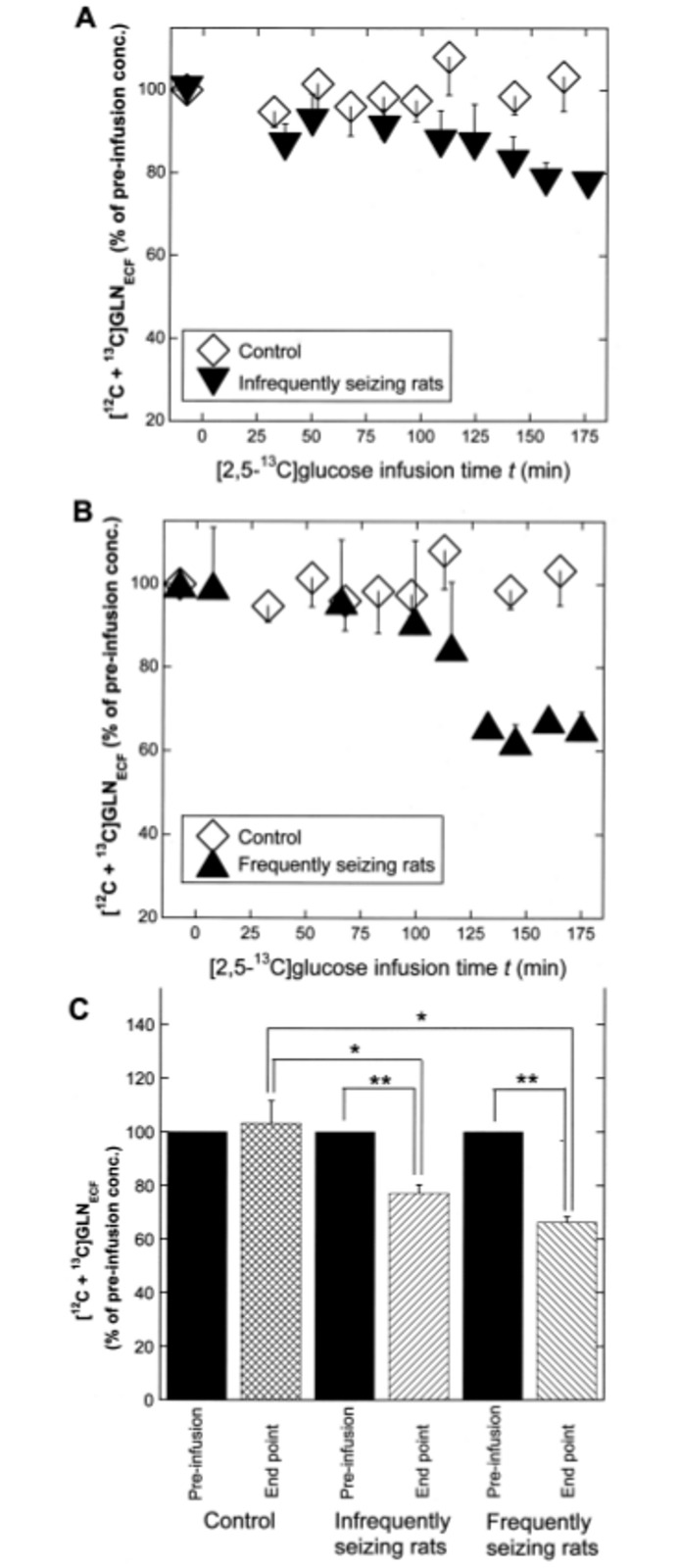
The time course of total [^12^C + ^13^C]GLN_ECF_ and comparison of the pre-infusion and the endpoint concentrations. The time course was expressed as the percentage of the pre-infusion concentration in each rat and the mean ± SE taken for each group. **A.** In infrequently seizing rats (inverted black triangle), the concentration decreased significantly while controls (open diamond) showed little change. **B**. Time course for frequently seizing rats (black triangle), showing a more pronounced decrease. **C**. A bar graph comparing the pre-infusion and the endpoint [^12^C + ^13^C} GLN_ECF_ in the control, the infrequently-seizing and the frequently-seizing rats. ** *p* < 0.01, * *p* < 0.05. (see text).

As shown in Table 1, the concentration of dialysate GLN before infusion was 29.9 ± 3.5 μM in controls, 36.4 ± 3.0 μM in infrequently seizing rats, and 29.5 ± 2.7 μM in frequently seizing rats, with no statistically significant difference among them. Fig 7 shows the time course of total [^12^C+^13^C]GLN_ECF_ during [2,5-^13^C]glucose infusion in the three groups. [^12^C+^13^C]GLN_ECF_ was expressed as the percentage of the pre-infusion concentration in each rat, and the mean ± SE was taken for each group. Although ANOVA was performed on three groups simultaneously, the time-course data for groups II and III, compared to the control, are shown in separate panels to avoid overlap of symbols and error bars. As shown in Fig 7A, no significant change was observed in controls, but in infrequently seizing rats, GLN_ECF_ decreased at *t* = 176 min to 77.1 ± 3.2%. As shown in Fig 7B, frequently seizing rats showed a more pronounced decrease at *t* = 175 min to 66.2 ± 2.1%. Fig 7C compares, in a bar graph, the pre-infusion and the endpoint [^12^C + ^13^C]GLN_ECF_ concentrations (expressed as % of the pre-infusion concentration) in the control, infrequently-seizing and frequently-seizing rats. Within each group (infrequently- or frequently-seizing rats), the endpoint GLN_ECF_ was significantly lower than the pre-infusion concentration with *p* < 0.01 (**). Among groups, the endpoint GLN_ECF_ in the infrequently seizing rats was significantly lower than in controls with *p* < 0.05 (F = 10.3; Tukey’s HSD test). The endpoint GLN_ECF_ in the frequently seizing rats was also significantly lower than in control with *p* < 0.05 (F = 10.3; Scheffe’s test).

## Discussion

### Seizure frequency and probe location

The difference in the mean number of seizures between infrequently seizing rats (group II) and frequently seizing rats (group III) (Table 1) is due to spontaneous nature of chronic-phase recurrent seizures [12–14]. The procedure for determining the locations of EEG electrodes and microdialysis probes in this study is the same as that used in our previous publications [14, 26, 28]. As shown in Table 1, the locations of EEG electrodes and microdialysis probes were virtually the same in all three groups, so the observed differences in (a) the time course of ^13^C enrichment and (b) the total [^12^C + ^13^C] concentration of GLU_ECF_ and GLN_ECF_ reported in this study were not due to any difference in electrode or probe location and can reasonably be attributed to differences in seizure activity, as described below.

### Frequent seizures induced faster flux of GLU from the neuron to ECF and triggered a chain reaction of excitation

This study showed that the rate of ^13^C enrichment of GLU_ECF_ C5 during *t* = 0–120 min of [2,5-^13^C]glucose infusion in rats undergoing frequent seizures was significantly higher than in controls or in those with infrequent seizures (Fig 3C and Table 2). These KA rats also showed gradual elevation of [^12^C+^13^C]GLU_ECF_ between *t* = 80–120 min and a significant elevation between *t* = 125–167 min (Fig 4). The elevation was probably caused by synchronous firing of glutamatergic neurons, such as is observed in the ictal discharge of Fig 1A (top trace). It is worth mentioning that the fractional ^13^C *enrichment* of GLU_ECF_ C5 measured by GCMS (Figs 2 and 3 and Table 2) is quite independent of the total [^12^C+^13^C]GLU_ECF_ *concentration* measured by HPLC (Fig 4).

These findings suggest the following sequence of events. The significantly higher rate of ^13^C enrichment of GLU_ECF_ C5 in frequently seizing rats suggests that exocytosis of neurotransmitter glutamate from pyramidal neurons to synaptic fluid occurred at a rate faster than that of the glial uptake of GLU_ECF_ and resulted in the gradual accumulation of GLU_ECF_ *(t* = 80–120 min). This, in turn, overstimulated GLU receptors in the CA3 region, which is highly populated with glutamatergic neurons with recurrent networks [38]. This is likely to have induced a chain reaction of excitation, which recruited a large population of neurons into epileptic discharges (Fig 1A), resulting in further elevation of GLU_ECF_ (Fig 4, *t* = 125–167 min).

In normal rats, the neurotransmitter GLU is cleared from ECF mainly by glial uptake transporter EAAT2 [15] [20] (S1 Fig), so it is important to consider whether impairment of this transport could have played a role in the observed elevation of GLU_ECF_. Another proposed explanation for GLU_ECF_ elevation is the reduction of glutamine synthetase (GS) observed in the hippocampal formation of mesial temporal lobe epilepsy patients [39]. This can lead to the accumulation of glial GLU and reduced glial uptake of GLU_ECF_. [40]. Previous reports on the EAAT2 and GS levels in the chronic phase of KA rats are described later in this section. In the present study, there was no statistically significant difference in the pre-infusion concentrations of dialysate GLU among the three groups, although the concentration was highest in the frequently seizing rats (Table 1 and Fig 4). This observation, combined with the higher flux of GLU as measured by the rate of ^13^C enrichment of GLU_ECF_ C5 (Table 2), suggests that the *primary* cause of GLU_ECF_ accumulation observed in KA rats of this study was the seizure-induced increased exocytosis of GLU to ECF rather than the impairment of glial uptake.

In rats undergoing intermittent and infrequent seizures, the rate and the time course of ^13^C enrichment of GLU_ECF_ C5 (Fig 3B and 3C and Table 2) and the time-course of total GLU_ECF_ (Fig 4) were similar to those in controls. A likely explanation is that when seizures are rare, the flux of GLU from the neuron to ECF returns to near-normal levels in the inter-ictal period. To detect these short time changes, ^13^C enrichment analyses at higher temporal resolution will be needed, as discussed in Section **MS methodology—advantages, limitations and potential for improvement.** Likewise, the similarity of the time-courses of total GLU_ECF_ between infrequently seizing and control rats can most reasonably be attributed to the reversal of elevation of total GLU_ECF_ during quiescent inter-ictal period, which was reported in our previous study [14] and is described below.

It is informative to ask why the GLU_ECF_ concentration measured before the start of the 3-h [2,5-^13^C]glucose infusion (Table 1) is not significantly higher in the frequently seizing rats compared to those with rare seizures and controls. A higher concentration is expected if the seizure patterns are the same before and during the infusion. Chronic-phase recurrent seizures occur spontaneously [12, 13] and the time of occurrence is unpredictable on a short time scale. Our previous study reported EEG recording of seizures in combination with analyses of changes in GLU_ECF_ in the same KA rat model for 5 h [14]. As shown in Fig 5a, 5b and 5c of that study, the elevation of GLU_ECF_ does correlate with the frequency and magnitude of seizure activity, but the pattern of seizure activity during the first 2.5 h is not the same as in the subsequent 2.5 h. In this earlier study too, the basal GLU_ECF_ concentration measured before the start of the experiment was 1.67 ± 0.32 μM in KA rats that showed frequent seizures in the subsequent 5 h and 1.5 ± 0.18 μM in those that did not (Table 2). Furthermore, this previous study showed that when seizures cease, GLU_ECF_ decreases slowly from the elevated level at *t* > 265 min in R1039 (Fig 5a) and *t* > 275 min in R1035 (Fig 5b). This reversal of GLU_ECF_ elevation during quiescent inter-ictal period accounts, at least in part, for the observation that basal GLU_ECF_ concentration is not significantly different between KA rats that subsequently showed frequent seizures and those that did not. In the present study, the classification of frequently seizing and infrequently seizing rats in Table 1 is based on the number of seizures observed during the 3-h glucose infusion because the focus of this paper is to examine the possible effect of frequent seizures on the rate of ^13^C enrichment of GLU_ECF_ in addition to examining their effect on total GLU_ECF_.

This study does not examine possible impact of glia and cerebrovascular mechanisms of glutamate homeostasis on our results, so it is useful to outline what is known and point out areas for future investigation.

For the impact of glia, an important issue is whether the glial glutamate transporter EAAT2, which plays a major role in the clearance of neurotransmitter GLU from the synaptic fluid, is impaired in the chronic-phase of KA rats. A previous study [41] reported that rats given unilateral amygdalar injection of KA (0.5 μg) developed spontaneous secondarily generalized seizures and, at 60 days post KA, EAAT2 (both mRNA and the transporter protein) were reduced by ~50%. This suggests a down-regulation of EAAT2 in the chronic phase. However, their dose of KA (0.5 μg) which induced secondarily generalized seizures in the chronic phase is higher than ours (~0.3 μg) which induced mainly electrographic seizures. Measurement of the EAAT2 level by Western blotting in our KA rats is an important area for future investigation.

A major glutamate-metabolizing enzyme in astrocytes is glutamine synthetase (GS). In rats given intraperitoneal injection of KA (10 mg/kg), GS concentration in the hippocampus, measured by semi-quantitative Western blotting, did not differ from the control at 11 weeks post KA [42]. Accordingly, it is unlikely that alteration in GS activity contributed to the elevation of GLU_ECF_ or to the increased ^13^C enrichment of GLU_ECF_ C5 in response to frequent seizures reported in this study.

An astrocytic enzyme that has been implicated in modulation of chronic-phase seizures is adenosine kinase. This enzyme regulates the level of brain endogenous adenosine, which is a potent natural anticonvulsant that contributes to seizure suppression [43]. In mouse hippocampi lesioned by low-dose unilateral kainate injection, adenosine kinase activity increased by 177% during chronic-phase seizures, suggesting that overexpression of adenosine kinase contributes to seizure progression by reducing endogenous adenosine. However, enhanced immunoreactivity to the enzyme was observed in the dentate gyrus and other hippocampal subregions but not in the CA3. Furthermore, a recent report [44] showed that a reduction (by 94–96%) of adenosine kinase expression in the rat hippocampus provided protection against kainate-induced neurodegeneration in the dentate hilar region but not in the CA3 region. Thus, while these are important findings, it is open to question whether the chronic seizures recorded from the CA3 region of our KA rats were modulated by adenosine kinase activity.

Intrahippocampal kainate injection elicits secretion of pro-inflammatory cytokines, notably interleukin1β by microglia of M1 phenotype; interleukin 1β concentration in the injected hippocampus was elevated 16-fold 24 h after KA injection [45]. Such inflammatory response promotes reactive astrogliosis, which persisted in the CA3 region even 30 days after KA injection when measured by immunoreactivity to the astrocyte marker, viz glial fibrillary acidic protein [46]. Neuroinflammation by the M1 microglia is sometimes accompanied by an upregulation of inducible nitric oxide synthase ([47] and references cited therein). In one kainate model [48], the production of nitric oxide in the hippocampus peaked at 8 h after status epilepticus and the elevated levels persisted for 6 weeks. In another kainate model [47], a 3-day treatment with a selective inhibitor of nitric oxide synthase starting 4 h after the status epilepticus reduced significantly (>90%) the occurrence of spontaneous recurrent seizures in the subsequent 6-month period, as monitored by cortical electrodes. Taken together, the results suggest that nitric oxide production in the early stage of epileptogenesis contributes to the development of chronic-phase seizures. However, the systemic KA doses in those two studies (11 mg/kg subcutaneous [48] or repeated intraperitoneal injections totaling >12.5 mg/kg [47] induced chronic-phase seizures significantly more severe than the recurrent seizures, which were mainly electrographic, induced by the low-dose unilateral intrahippocampal injection (~0.3 μg) in our KA rats. Accordingly, we must await future investigation to clarify possible roles of pro-inflammatory cytokines and upregulation of inducible nitric oxide synthase in modulating the electrographic chronic-phase seizures reported in this study.

With respect to the impact of cerebrovascular mechanisms on glutamate homeostasis, the following information is available from literature. The brain uptake index of GLU is 3.21 ± 0.26 compared to 100 for water [49], and is one of the lowest among amino acids. The permeability of GLU across the blood-brain barrier is ~1/30^th^ of that of glucose [50] in the normal brain. Accordingly, upon intravenous infusion of ^13^C-glucose, brain GLU is ^13^C-enriched predominantly by the ^13^C-glucose taken up into the brain. A relevant question is whether the blood-brain barrier to GLU is impaired in the hippocampus of kainate-treated rat during the chronic phase (~7–8 weeks post KA) when the present study was performed. An influx of blood ^13^C-glutamate (if any) into the brain can affect the ^13^C enrichment time-courses of brain GLU_ECF_ reported in the present study. A recent study [51] induced severe seizures for ≥ 4h in rats by i.p. injection of kainate (14 mg/kg followed by 7 mg/kg), and examined the blood-brain barrier leakage to gadobutrol-containing water by T1 mapping. The gadolinium leakage rate in the hippocampus was ~3 times the pre-seizure level at day 1, but by 6 weeks, was only ~1.5 times the pre-seizure level, indicating that the blood-brain barrier permeability to water is partially restored in the chronic phase. The kainate dose and the duration of severe seizures in this longitudinal study are considerably higher than those in our study. It is also necessary to consider how the blood-brain barrier leakage to gadobutrol-containing water impacts the passage of glutamate from the blood to the brain. Useful information is provided in an earlier study [52] which examined the brain uptake of plasma α-[^14^C]aminoisobutyric acid, a non-metabolizable amino acid with a brain uptake index of 3.3 ± 0.68, which is very close to that of [^14^C]glutamate (3.21 ± 0.26) [49], after treatments with various doses of kainate. Six rats which received KA injection of 12 mg/kg or 7 mg/kg initially showed severe seizures which subsided within 24 h. By 3–7 days post KA, the blood-brain barrier permeability coefficient for α-[^14^C]aminoisobutyric acid in the hippocampal CA3 and CA1 regions were not significantly different from the control [52]. This report strongly suggests that in rats treated with a moderate dose of KA that induced short-term acute seizures like our rats, the brain uptake of glutamate was not significantly different from the untreated control even during the acute phase (3–7 days after KA). Because the longitudinal study [51] showed that blood-brain barrier leakage to water observed in the acute phase is significantly attenuated 6 weeks later, it is reasonable to assume that passage of blood glutamate into the brain was negligibly small in the chronic phase of our KA rats. Nevertheless, autoradiographic studies of our chronic-phase KA rats after injection of α-[^14^C]aminoisobutyric acid or [^14^C]glutamate following the protocols of [52] and [50] respectively may provide further insight into this issue.

### GLN_ECF_—^13^C-enrichment and total concentration change

The present study showed that the time-courses of the ^13^C enrichments of GLN_ECF_ C5 were apparently similar among the three groups (Fig 6). However, as shown in Table 2, the mean rate of ^13^C enrichment in the frequently-seizing KA rats was significantly higher than the mean rate in the control. A possible explanation is the significantly higher ^13^C enrichment of GLU_ECF_ C5, which, upon uptake into glia, contributes to the substrate pool for glial glutamine synthesis before glutamine effluxes to the ECF. Other factors that can affect the ^13^C enrichment of GLN_ECF_ C5 are the relative contributions of glial GLU and GLU_ECF_ as substrates for glutamine synthesis, the concentration of glutamine synthetase [42], the proliferation of astrocytes [46] in KA rats and the possible regulation of the transporter that mediates glial glutamine efflux to the ECF (sodium-coupled neutral amino-acid transporter subtype 3) by coupling with glial glutamate transport [53]. Among these, published reports on the glutamine synthetase concentration and the time-course of astrogliosis in the chronic phase of KA rats are described in the previous section. Further studies are needed to address the other issues.

Our results in Fig 7 show that the total [^12^C + ^13^C]GLN_ECF_ concentration decreased significantly, during the 3-h experimental period, in infrequently seizing rats, and the decrease was more pronounced in frequently seizing rats. The results are in good agreement with our previous reports of a significant reduction of GLN_ECF_ (in 5 h) in response to seizures in KA rats [14, 26] and in response to epileptiform discharges induced by disinhibition in normal rats [28].

It is informative to consider why total GLN_ECF_ showed significant decrease only at later time points post-infusion (125–175 min). In our previous study on frequently-seizing KA rats that showed GLU_ECF_ elevation [14], a very similar time-course of GLN_ECF_ decrease was observed and was attributed to a combination of two factors. The first factor is that an impaired glial uptake of GLU_ECF_, suggested by its observed elevation, decreased the availability of the substrate GLU for GLN synthesis and decreased glial GLN concentration. Decreased glial GLN concentration can cause decrease in GLN_ECF_, because SNAT3-mediated release of glial GLN into ECF (S1 Fig) depends on GLN_in_/GLN_o_ ratio and the rate of release decreases when the ratio falls significantly below the normal ratio of 20 [24, 54, 55]. In the present study, GLU_ECF_ elevation in the frequently-seizing KA rats was prominent at later time points 125–175 min as shown in Fig 4. This is likely to have contributed to the observed decrease in total GLN_ECF_ at this later time point (Fig 7B) by the sequence of events described above. The second factor that can contribute to the observed decrease of GLN_ECF_ is a faster rate of GLN_ECF_ uptake into neurons mediated by SNAT1/SNAT2 (S1 Fig) in response to epileptiform activity, because the concentration of GLN_ECF_ depends on the rate of uptake into neurons relative to the rate of release from glia. Evidence from our and other laboratories strongly suggests that GLN_ECF_, taken up into neurons and hydrolyzed to GLU (S1 Fig) contributes to the maintenance of neurotransmitter GLU pool during epileptiform activity ([26] and references cited therein). This factor can account for the observation in the present study that GLN_ECF_ decreases significantly in infrequently-seizing KA rats as well (Fig 7A), although to a lesser extent than in the frequently-seizing rats (Fig 7B).

### MS methodology—Advantages, limitations and potential for improvement

The concentration of GLU_ECF_ at the basal level is approximately 1/2,000^th^ of that of intracellular GLU [20]. Therefore, for ^13^C enrichment analysis, we used mass spectrometry, which is substantially more sensitive than NMR. This permitted ^13^C enrichment analyses of ~10 pmol of *t*BDMS-GLU at 10–15 min temporal resolution when GLU_ECF_ was collected from a hippocampal volume ~700 μm in diameter and 2 mm in length. A higher temporal resolution (e.g., 5 min) of ^13^C enrichment analysis is desirable to examine whether ^13^C enrichment returns to near-normal levels in the inter-ictal periods. For GLN_ECF_, the basal concentration was approximately 15- to 38-fold higher than that of GLU_ECF_ (Table 1), so the measurement of ^13^C enrichment at a higher time resolution (every 3–5 min) is feasible. Increased temporal resolution for ^13^C-GLU_ECF_ analyses may be achieved by further improvement in the sensitivity of mass spectrometry. It may also be feasible in the larger human hippocampus where a 10-mm or a 70-mm microdialysis probe has been used to collect extracellular neurochemicals (without ^13^C enrichment) [5]; with such probes, the quantity of GLU_ECF_ collected per min would be significantly higher, permitting the enhanced temporal resolution of ^13^C enrichment analysis by MS.

For ^13^C enrichment, this study used [2,5-^13^C]glucose, which has the advantage of ^13^C enriching GLU and GLN predominantly at C5 and to a lesser extent at C1. However, low ^13^C enrichment at C2, C3 and C4 occurred due to label scrambling during long infusion [35, 56, 57] and their contributions to the ^13^C enrichment of ion pairs m/z 330/331 and m/z 273/272 of *t*BDMS-GLU (which contain C2 to C5 of GLU) must be subtracted to obtain ^13^C-enrichment at GLU_ECF_ C5. Likewise, the contribution from ^13^C enrichment at C2, C3 and C4 of glutamine must be subtracted from the enrichment of the ion pairs m/z 198/199 and m/z 272/273 of glutamine-derived *t*BDMS-pyroGLU, which contain C2-C5 of glutamine, to obtain the ^13^C enrichment at GLN_ECF_ C5 (note that C1 is not contained in these ion fragments (Figs 2 and 5)). This was achieved by measuring the distribution of ^13^C labeling among the carbons of GLU and GLN in perchloric acid extracts of brains undergoing identical [2,5-^13^C]glucose infusion protocol by ^13^C NMR (S1 Table). Our results are in agreement with in vivo MRS results from the human brain [56] which showed that ^13^C enrichment at C2, C3 and C4 was less than 0.76% for GLU and less than 1.5% for GLN after 2 h of [2-^13^C]glucose infusion (for comparison with the present study, the fact that [2,5-^13^C]glucose infusion achieves an enrichment 2-fold higher than [2-^13^C]glucose infusion has been taken into account). The distributions of ^13^C labeling among C2, C3, C4 and C5 of GLU and GLN were expected to be the same in all rat groups studied here. Therefore, the observed differences in the time course and the rate of ^13^C enrichment of GLU_ECF_ C5 between frequently seizing rats and controls (Fig 3 and Table 2) are valid.

### Effects of hippocampal sclerosis on ^13^C enrichment of intracellular and extracellular GLU

Petroff et al. [16] reported that the rate of glutamate/glutamine cycling relative to the TCA cycle is reduced in sclerotic hippocampal tissue resected in temporal-lobe epilepsy patients after 4.3 h of [2-^13^C]glucose infusion. The results were based on ^13^C NMR analyses of intracellular GLU and other neurochemicals in the perchloric acid extract of the resected hippocampus. Another group reported increased glutamine synthesis compared with glutamate formation in cortical areas with sustained epileptiform activity in patients after the oral administration of [1-^13^C]glucose [58]. In a KA rat model, Alvestad et al [18] reported that the concentration of [4-^13^C]GLU in hippocampal formation 15 min after i.p. injection of [1-^13^C]glucose is significantly reduced in the sclerotic hippocampus compared to controls. This result is understandable when the concentration of [4-^13^C]GLU is expressed in μmol/g of hippocampal formation, which includes sclerotic tissue. The studies by Petroff *et a*l. and Alvestad *et al*. cited above provide valuable information on the effect of hippocampal sclerosis, which is common in human temporal lobe epilepsy and in the KA model, on the ^13^C labeling of hippocampal GLU, which is predominantly *intracellular*. The present study focuses on the rate of ^13^C enrichment of *extracellular* GLU C5, and does not address the question of how this may be affected by hippocampal sclerosis or neuronal loss. Among numerous histological studies on neuronal loss in the CA3 and CA1 regions of rat hippocampus following unilateral [12] [59] or bilateral [3, 10] kainate injection, the most detailed is the dose-dependent study of Magloczky and Freund [59] who reported that, at a kainate dose of 0.25 μg, which is very close to the dose of 0.31 μg (in 250 g rat) in the present study, neuronal degeneration in the kainate-injected CA3 was greater than 50% in the majority of rats (4 out of 7), and the degeneration in CA1 was between 10% to >50% in 5 out of 7 rats. Phelps et al. [60] reported short- and long-term histological changes to synaptic ultrastructure of rat hippocampus following intracerebroventricular injection of 0.5 μg kainate (which induced extensive selective neuronal loss very similar to that resulting from the intrahippocampal injection of KA described above). These investigators showed that, despite reactive synaptogenesis, the number of asymmetric synapses in the CA1 field was reduced (79% of control) at 14 weeks post-KA, and damaged myelin sheaths were still in evidence at 14 and 24 weeks post-KA. In KA treated rats as well as in other rodent models of epilepsy, reactive synaptogenesis can take the form of aberrant axonal sprouting, notably mossy fiber sprouting observed in the dentate gyrus [10, 12]. However, invasion of sprouting axons to CA3 region has not been reported in kainate-treated rats. In the CA1 region, collateral axonal growth of pyramidal neurons as well as spontaneous paroxysmal discharges in the somata and dendrites were reported in hippocampal slices prepared from chronic-phase rats that had been given intracerebroventiricular injection of 0.5 μg kainate [61]. However, in similarly treated rats 30 days post-KA, axonal growth into denervated CA1 area was not observed histologically [62]. Because mossy fiber sprouting creates recurrent excitatory circuity in the dentate gyrus [63], a relevant question is whether this aberrant circuit contributed to seizure activity recorded in the CA3 region in the present study. However, the sprouting density of dentate mossy fiber correlated with the number of behavioral seizures, but not with the number of electrographic seizures without behavioral components [12]. Because the majority of seizures observed in the present study are hypersynchronous-onset seizures without behavioral components (Section **EEG characteristics of KA rats**), it is unlikely that mossy fiber sprouting within the dentate gyrus contributed to the glutamatergic population bursts observed in the present study. While parallel histological study, in addition to those already reported, is likely to be informative, this is beyond the scope of the present pilot study. I have adopted a reasonable assumption that while a sizable neuronal population was probably degenerate in the KA rats of this study, ^13^C labelling of GLU and its release to ECF occurred in surviving or re-innervated pyramidal neurons with intact or functional pyruvate dehydrogenase/tricarboxylic acid pathways and the exocytosis mechanism.

### Clinical relevance and future perspective

Microdialysis studies in temporal lobe epilepsy patients have provided valuable insight into the correlation of ictal and inter-ictal GLU_ECF_ levels with (a) the severity of spontaneous seizures and (b) hippocampal sclerosis in both conscious [1, 3–6] and anesthetized [64, 65] patients. However, it is understandable that many recent clinical microdialysis studies are performed when patients are resting quietly and when seizures are under control by the use of anti-epileptic drugs. Animal models of temporal lobe epilepsy permit the examination of the correlation of epileptic activity with changes in the concentration of extracellular neurochemicals during awake ictal periods in the absence of anti-seizure treatment. Using ^13^C labeling and the analysis of ^13^C enrichment by GCMS at a 10 picomol level of GLU_ECF_, this study shows for the first time that the flux of GLU from the neuron to ECF is increased during frequent seizures in vivo. It is hoped that the advantages, limitations and the potential for improvement of this novel approach presented in this study will be useful in designing future ^13^C labeling studies of extracellular neurochemicals in pre-clinical or clinical settings.

## Supporting information

S1 Table Distribution of ^13^C in C2-C5 of GLU and GLN after intravenous infusion of [2,5-^13^C]glucose as measured in brain extracts by ^13^C NMR. (DOCX)

S1 Fig A schematic diagram of the glutamine-glutamate cycle showing major metabolic pathways of Glutamate (GLU) and Glutamine (GLN) and their transport pathways between the synaptic vesicle, Extracellular Fluid (ECF), glia and the neuron. α-KG, α-ketoglutarate; EAAT2, excitatory amino acid transporter subtype 2; GLC, glucose; GLU_gl_, glial GLU derived from glucose by the tricarboxylic acid cycle; GLU_NT_, neurotransmitter GLU; GLNase, glutaminase; GS, glutamine synthetase; SNAT1,2,3, sodium-coupled neutral amino acid transporter subtypes 1,2,3 (adapted from Kanamori & Ross 2011 [14] with permission).
(TIF)

S2 Fig Location of the EEG recording electrode and the microdialysis probe in a coronal map of rat brain at AP = - 5.6 mm. The electrode tip is in the CA3 and the microdialysis probe in the CA1/CA3 region of the hippocampus. REC: EEG recorder (reproduced from Kanamori 2015 [28] with permission).
(TIF)

S3 Fig Increase in the ratio of ^13^C enrichment at C2+C3+C4 to ^13^C enrichment at C2+C3+C4+C5 for (A) GLU and (B) GLN. (TIF)

S4 Fig The structures of *t*BDMS-pyroGLU (M+:m/z 357), and of its fragment ions m/z 300, m/z 272 and m/z 198 as described by [32]. (TIF)

## Abbreviations

EAAT2: excitatory amino acid transporter subtype 2
GCMS: gas-chromatography mass-spectrometry
GLN_ECF_: extracellular glutamine
GLU_ECF_: extracellular glutamate
GS: glutamine synthetase
KA: kainic acid
MTBSTFA: *N*-Methyl-*N*-(*t*-butyldimethylsilyl)-trifluoroacetamide
SNAT1,2,3: sodium-coupled neutral amino acid transporter subtypes 1,2,3
*t*BDMS: *t*-butyldimethylsilyl
