# Supplementary material for: Faster flux of neurotransmitter glutamate during seizure — Evidence from 13C-enrichment of extracellular glutamate in kainate rat model
Source: PLoS One. 2017 Apr 12;12(4):e0174845. doi: 10.1371/journal.pone.0174845 (PMC5389799; doi:10.1371/journal.pone.0174845)
Supplement: S1 Table — (DOCX) [file pone.0174845.s001.docx]

**Supplementary Table 1. Distribution of ^13^C in C2-C5 of GLU and GLN after intravenous infusion of [2,5-^13^C]glucose as measured in brain extracts by ^13^C NMR**

| **Metabolite** | **Infusion time (h)** | **Fractional ^13^C enrichment** | | | | **Enrichment (C2+C3+C4)/**  **Enrichment (C2+C3+C4+C5)** |
| --- | --- | --- | --- | --- | --- | --- |
|  |  | **C2** | **C3** | **C4** | **C5** |  |
| GLU | 2.0 | 0.02*^a^* | 0.018*^a^* | N.D. | 0.35*^a^* | 0.038/0.388 = 0.098 |
|  | 3.6 | 0.046*^b^* | 0.046*^b^* | 0.019*^c^* | 0.45*^c^* | 0.111/0.561= 0.198 |
| GLN | 2.0 | 0.0215*^a^* | 0.029*^a^* | N.D. | 0.21*^a^* | 0.0505/0.2605=0.194 |
|  | 3.6 | 0.046*^c^* | 0.058*^c^* | N.D. | 0.33*^c^* | 0.104/0.434=0.239 |
|  |  |  |  |  |  |  |

1. Kanamori and Ross, 2001 [29]
2. Kondrat *et al*. (2002) [21] Table 2
3. Unpublished results from our laboratory based on NMR data from the same brain extracts as in the above two publications.

ND: not detected
