# Supplementary figures and images for: Faster flux of neurotransmitter glutamate during seizure — Evidence from 13C-enrichment of extracellular glutamate in kainate rat model
Source: PLoS One. 2017 Apr 12;12(4):e0174845. doi: 10.1371/journal.pone.0174845 (PMC5389799; doi:10.1371/journal.pone.0174845)

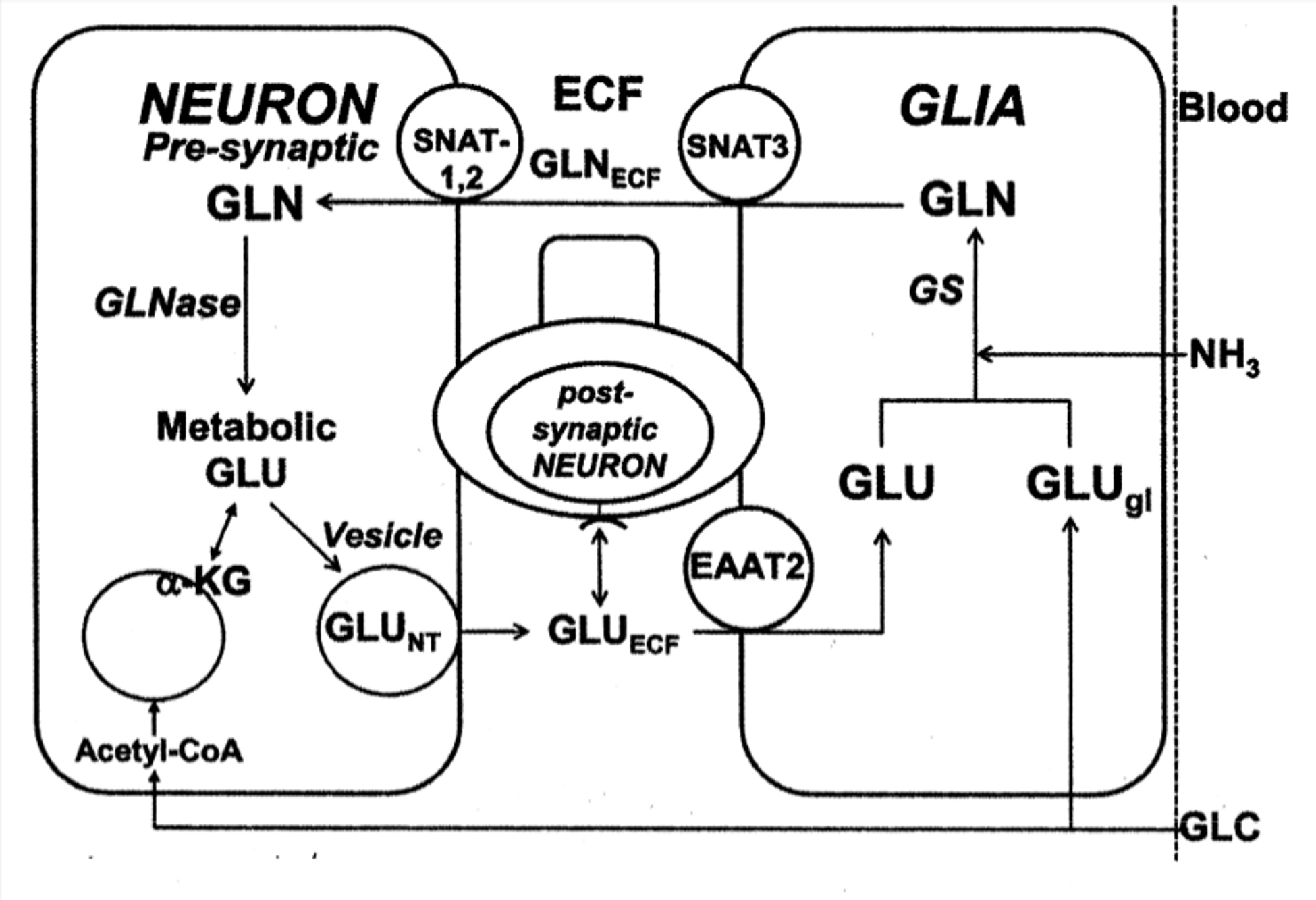

Supplement: S1 Fig — α-KG, α-ketoglutarate; EAAT2, excitatory amino acid transporter subtype 2; GLC, glucose; GLUgl, glial GLU derived from glucose by the tricarboxylic acid cycle; GLUNT, neurotransmitter GLU; GLNase, glutaminase; GS, glutamine synthetase; SNAT1,2,3, sodium-coupled neutral amino acid transporter subtypes 1,2,3 (adapted from Kanamori & Ross 2011 [14] with permission). (TIF) [file pone.0174845.s002.tif]

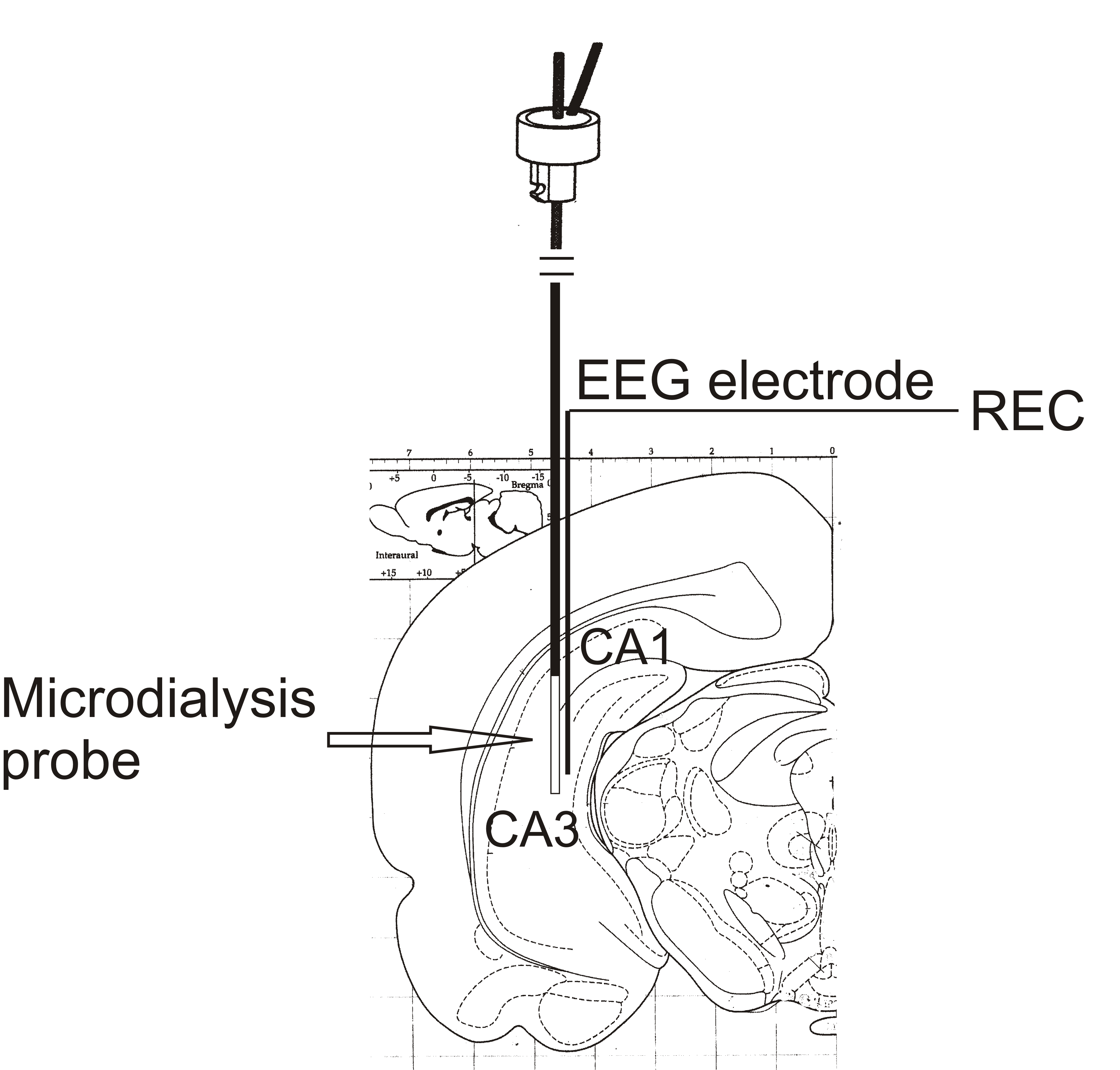

Supplement: S2 Fig — The electrode tip is in the CA3 and the microdialysis probe in the CA1/CA3 region of the hippocampus. REC: EEG recorder (reproduced from Kanamori 2015 [28] with permission). (TIF) [file pone.0174845.s003.TIF]

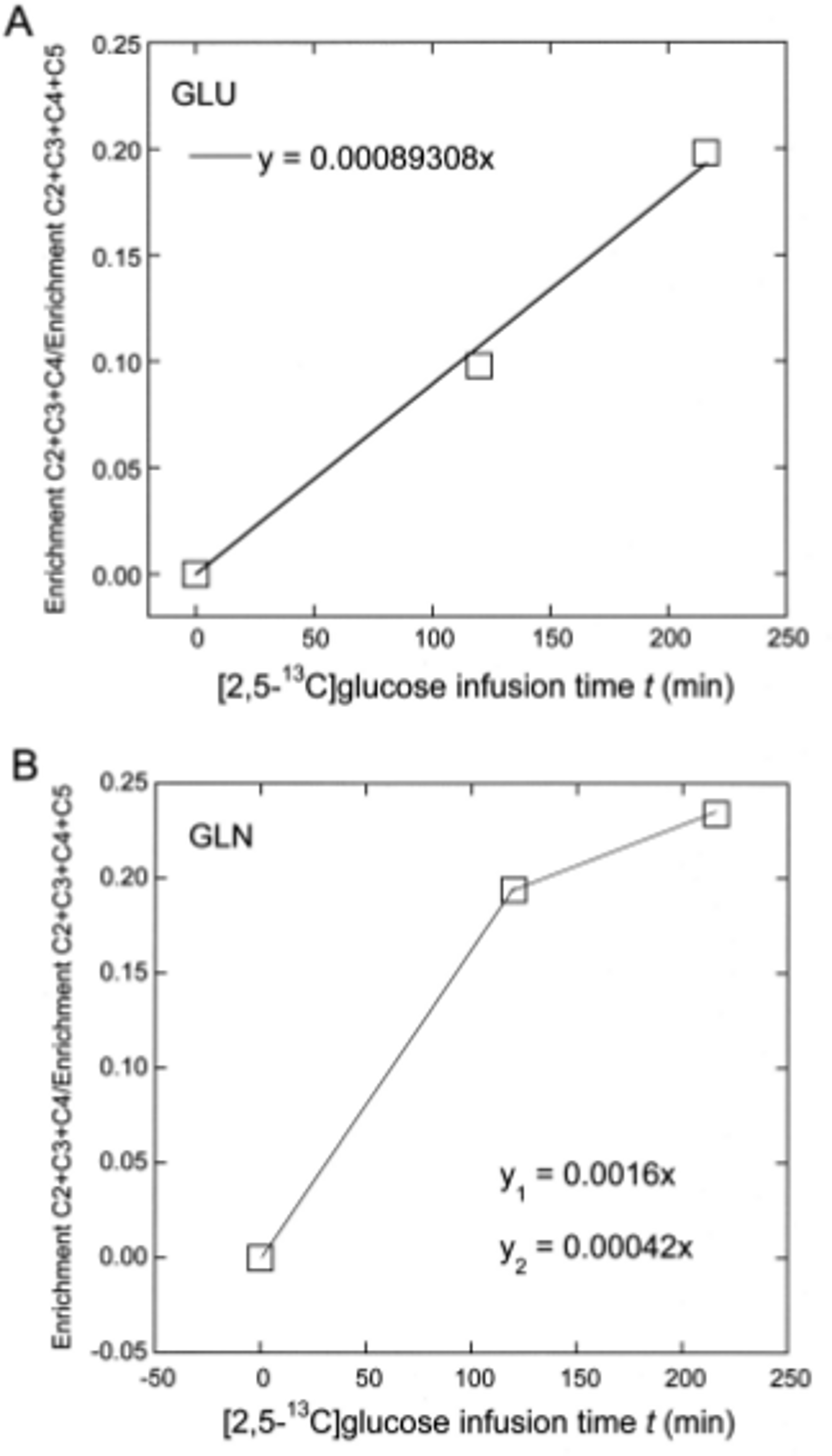

Supplement: S3 Fig — (TIF) [file pone.0174845.s004.tif]

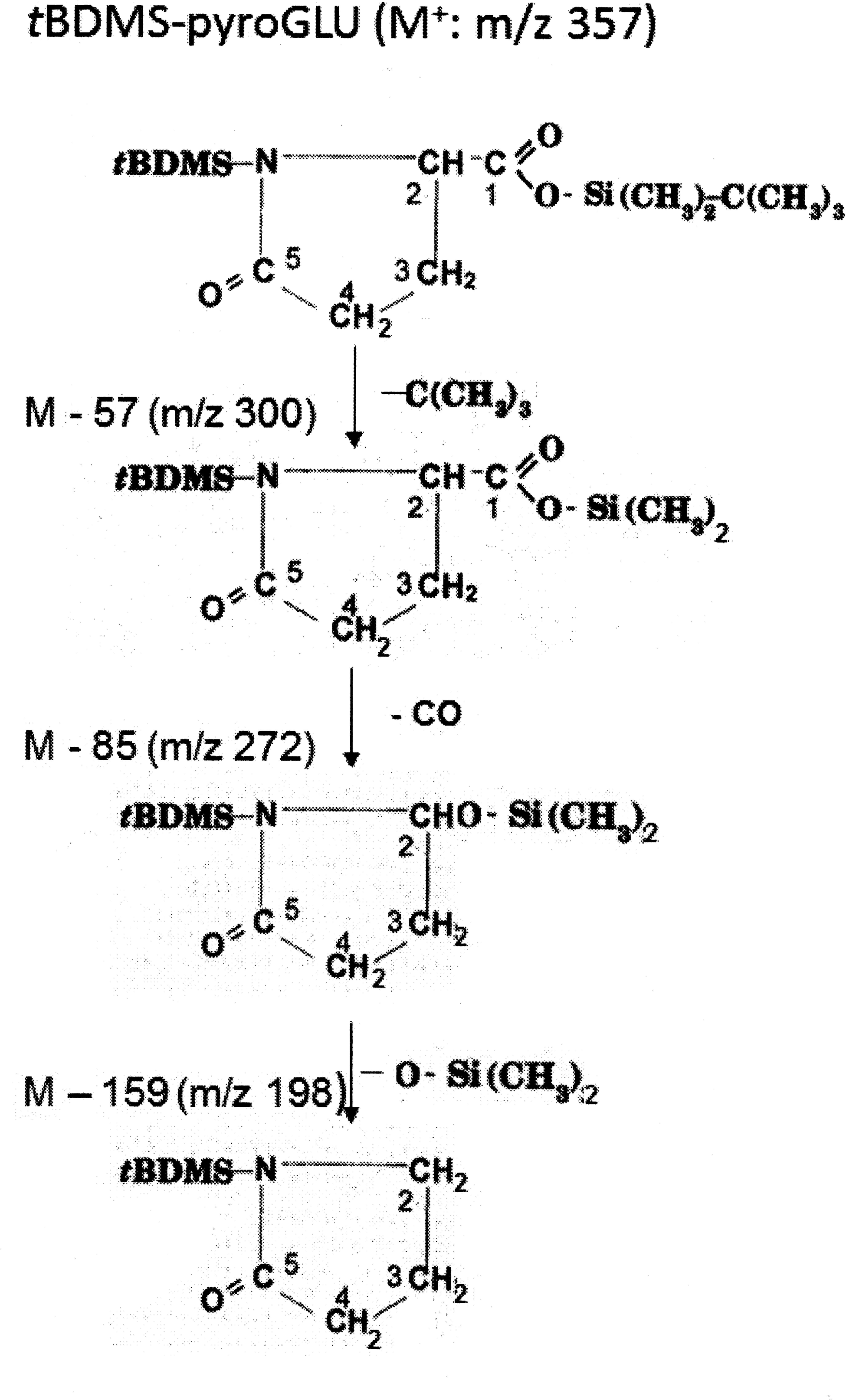

Supplement: S4 Fig — (TIF) [file pone.0174845.s005.tif]
